# Light-Matter Interaction in Ultrastable Tunneling Nanogaps

**DOI:** 10.1021/acsnano.5c03217

**Published:** 2025-07-23

**Authors:** Yuankai Tang, Saurav Prakash, Proloy Nandi, Ariando Ariando, Amit Agrawal, Hayk Harutyunyan

**Affiliations:** † Department of Physics, 1371 Emory University, Atlanta, Georgia 30322, United States; ‡ Department of Physics, 37580 National University of Singapore, Singapore 117551, Singapore; § Department of Engineering, 2152 University of Cambridge, Cambridge CB3 0FA, U.K.; ∥ Kyung Hee University, 26 Kyungheedae-ro, Dongdaemun-gu, Seoul 02447, Korea

**Keywords:** tunnel junctions, photoassisted tunneling, optical rectification, hot electrons, nanogaps

## Abstract

Light emission and detection through tunnel junctions have emerged as a promising platform for studying nanoscale light–matter interactions, including electroluminescence and photoassisted transport. However, controlling these interactions in the tunneling regime has been challenging due to complex underlying mechanisms that remain poorly understood. A major obstacle is the difficulty in forming stable junctions that can function reliably over extended periods. In this study, we fabricate ultrastable tunneling junctions consisting of epitaxial indium–tin-oxide, epitaxial lutetium oxide, and gold. With their stable and consistent tunneling currents, we investigate photon-assisted transport phenomena using simple direct-current detection. Our results demonstrate that optical rectification is the primary contributor to the laser-induced current, alongside thermal effects and hot-electron currents. Furthermore, owing to their epitaxial nature and high breakdown threshold, this ultrastable platform holds promise for future real-world applications, including nanoscale light sources and multifunctional photodetectors.

## Graphical abstract

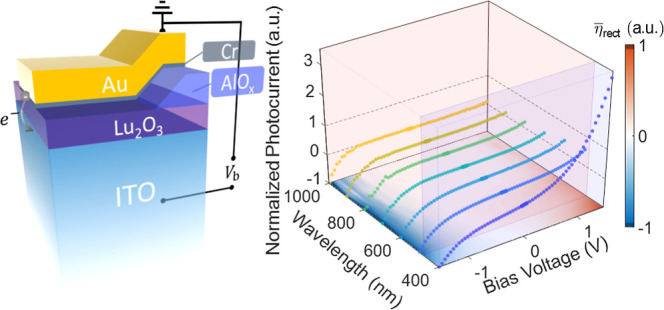

## 1 Introduction

In the early 20th century, the emergence of quantum theory unveiled a distinct realm, departing from the classical description of macroscopic and microscopic phenomena, and leading to groundbreaking discoveries that advanced our understanding of the universe.^1,2^ A key example is the observation of quantum tunneling,^3^ where an electron or hole in a metal or semiconductor can penetrate a potential barrier, representing one of the fundamental discoveries in quantum physics. Common structures that support electron tunneling include metal–insulator–metal (MIM) junctions,^4^ metal–insulator–semiconductor (MIS) junctions,^5^ and semiconductor junctions.^6^ This ability of charge carriers to tunnel through thin insulating barriers has enabled various promising electronic devices, such as tunneling diodes, switching diodes, resonant-tunneling diodes, and hot-electron transistors.^7^ Furthermore, the scanning tunneling microscope (STM), a powerful tool for observing and manipulating the atomic world, is also based on the phenomenon of electron tunneling.^8^

In addition to their unique electrical properties, tunneling junctions provide a compelling platform for studying light–matter interactions, encompassing phenomena such as electroluminescence (EL) and photoassisted transport,^9^ especially when engineered with plasmonic structures. The fundamental mechanism behind EL in these junctions is inelastic tunneling,^10–13^ where electrons lose a portion of their energy during the tunneling process and emit photons. Due to their potential as nanoscale light sources, numerous efforts have been made to enhance and control the EL process in tunneling junctions.^10,11,14–18^ These efforts include the use of optical antennas,^19,20^ resonant electron tunneling structures,^21^ and van der Waals materials.^22,23^ Furthermore, hot electrons generated during tunneling processes can also lead to above-threshold emission.^24–26^ In the context of photoassisted transport, several mechanisms can generate photocurrents, including optical rectification,^27–32^ hot carrier current,^33–36^ field emission current,^37,38^ and thermal effects.^31,39–43^ Typically, multiple mechanisms can operate simultaneously, complicating the analysis of photoassisted transport. For instance, demonstrating optical rectification in nanostructures is challenging not only because of ultraweak signals but also due to the presence of similar phenomena caused by other underlying physical mechanisms. It is essential to distinguish and harness these mechanisms for specific applications, such as near-field measurements,^28,29^ wavelength determination,^34^ and single-molecule detection.^44,45^ However, the limited breakdown voltage and ultrathin insulating layers in tunneling junctions make it difficult to maintain strong and stable tunneling currents for extended measurements, especially at room temperature. As a result, advanced techniques like lock-in detection are often required to detect and differentiate the subtle features of photoassisted transport, which are inherently limited by weak signals and the instability of tunneling junctions.

In this work, we successfully fabricate and characterize ultrastable tunnel junctions composed of a thin Au film on epitaxial Lu_2_O_3_, serving as the insulating tunnel barrier, grown on an epitaxial indium–tin-oxide (ITO) bottom electrode. Clear electroluminescence (EL) spectra are detected from the junction under applied bias voltages. Using pulsed laser excitation, we observe distinct photoassisted tunneling phenomena in these junctions across a broad wavelength range, from ultraviolet (UV) to near-infrared (NIR), specifically from 400 to 1350 nm. The exceptional stability of these junctions allows us to extract information on photocurrent, conductance, and conductance nonlinearity through straightforward direct current (DC) measurements and enables us to identify the dominant mechanism in each regime by systematically varying the insulating layer thickness, excitation power, and wavelength. Our experimental findings align with both classical and quantum descriptions of optical rectification, which plays a dominant role in the photoassisted process. When the incident photon energy is low enough (>700 nm), optical rectification is well described within the classical treatment. When the photon energy becomes high (<500 nm), however, deviations between classical and quantum descriptions become significant. A distinct feature of optical rectification, which can only be explained by the quantum description, is observed at 400 nm excitation. Additionally, hot electron transport and thermal effects influence the process, particularly at high absorption or short incident wavelengths (<700 nm). Thus, the ITO/Lu_2_O_3_/Au junctions represent a promising platform for studying light–matter interactions in the tunneling regime and hold significant potential for applications as on-chip optoelectronic devices, stable nanoscale light sources, and detectors.

## 2 Results

### 2.1 Tunneling Properties of ITO/Lu_2_O_3_/Au Junctions

According to quantum theory,^1,46^ free electrons in conductors with energy lower than the potential barrier of an adjacent material, typically an insulator, can undergo tunneling (Figure 1, gray arrows). The Wentzel-Kramers-Brillouin (WKB) approximation^1^ provides a method for calculating the general probability of transmission of an electron through a potential barrier, *T*, defined as *T* = *e* ^–2γ^, where γ = ∫_0_^Δ*s*^|*p*(*x*)|d*x* where Δ*s* represents the width of the barrier, and *p* is the momentum of the electron. Thus, the tunneling current density can be determined by combining the Fermi–Dirac distribution of electron energy with the probability equation.

**Figure 1:**
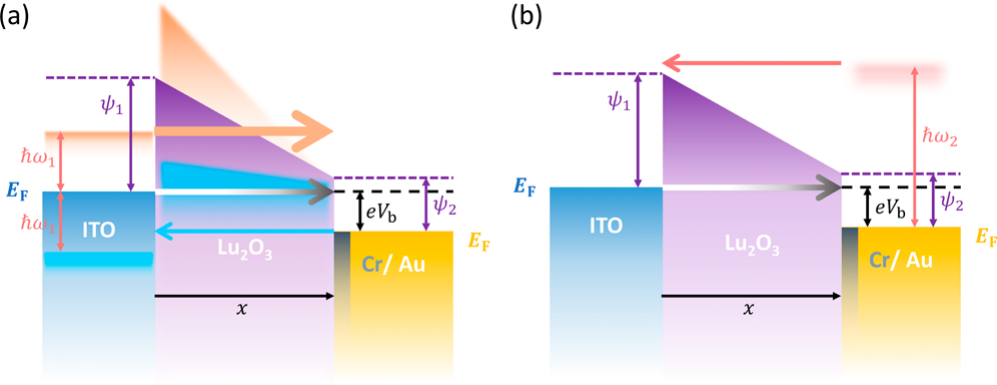
Schematic diagram of electron tunneling. Gray arrow indicates normal tunneling electrons, −*I*_dc_. (a) Schematic diagram of the optical rectification mechanism. Orange and blue arrows indicate tunneling electrons caused by light-induced fields. (b) Schematic diagram of hot electron current, −*I*_hot_ (internal photoemission process). Red arrow indicates hot electron flow.

Numerous theoretical studies have explored the formulation of tunneling current density, *J*, over the past few decades.^41,47–49^ For example, Simmons’s formula provides a well-approximated expression for *J*, based on the WKB method,^47^ expressed as

1 $$J=J_{0}\left[\varphi e^{-A\sqrt{\varphi }}-\left(\varphi +eV_{b}\right)e^{-A\sqrt{\varphi +eV_{b}}}\right]$$

where φ = ∫_0_^Δ*s*^ψ­(*x*)­d*x*/Δ*s* is the average potential barrier height, ψ­(*x*) is the barrier height along the *x*-axis, *e* is the charge of an electron, *J*_0_ = *e*/2π*h*(βΔ*s*)^2^, $A=4\pi \beta \Delta s/h\sqrt{2m}$ , β is the correction factor, Δ*s* is the width of the barrier, and *h* is the Planck constant. Therefore, the tunneling current *I*_dc_ can be expressed as *I*_dc_ = *C* × *J*, where *C* represents the cross-sectional area of the tunneling junction, and *J* is the net flow resulting from two opposing current flows originating from the two separated electrodes. Two critical factors, namely the barrier height ψ and the gap width *d*, determined by the constituent materials and geometrical structure of the junction, influence *J*. Reducing ψ and narrowing *d* can both contribute to an increase in *I*_dc_. It should be noted that usually *d* ≠ Δ*s*, because the latter can be affected by the bias voltage *V*_b_.

In this paper, we fabricate ultrastable ITO/Lu_2_O_3_/Au tunnel junctions composed of epitaxial layers of ITO and Lu_2_O_3_.^50^ The junction consists of two electrodes, ITO, and Au, separated by an ultrathin Lu_2_O_3_ insulating layer of the thickness *d* (Figure 2a). The closely matched lattice constants of YSZ (5.14 Å),^51^ ITO (10.12 Å),^52^ and Lu_2_O_3_ (10.40 Å),^53^ during the epitaxial growth process contribute to the long-term stability of the junction.^50^ The cross-sectional high-angle annular dark-field scanning transmission electron microscopy (HAADF-STEM) images and X-ray diffraction (XRD) demonstrate the high quality of the epitaxially grown films in the junction (Supporting Information, Section S15). Furthermore, the substantial bandgap of Lu_2_O_3_, approximately 5 eV, enables the application of higher bias voltages *V*_b_, which is advantageous for controlling the tunneling process, though it may reduce the direct current *I*_dc_.

**Figure 2:**
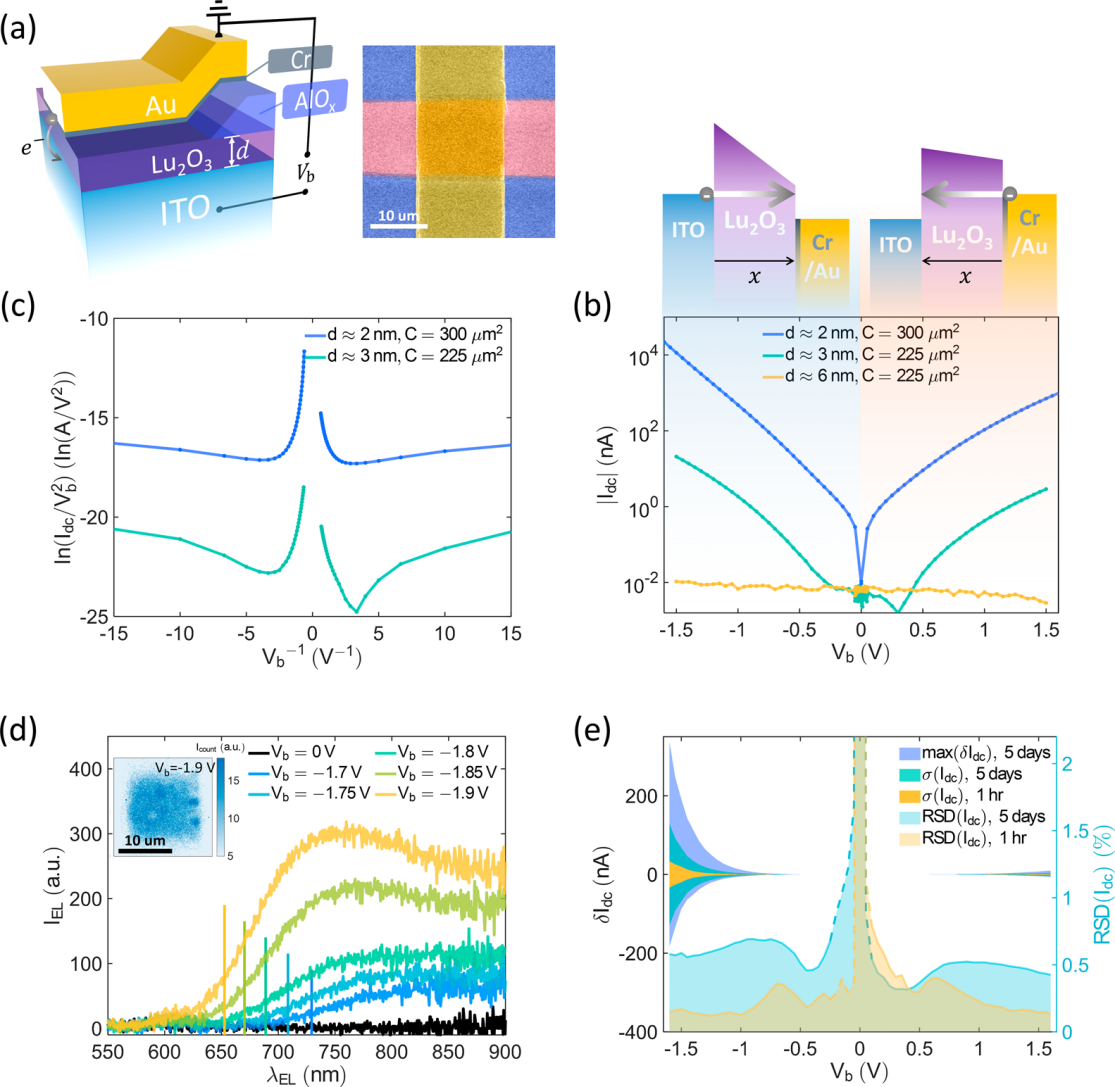
Properties of ITO/Lu_2_O_3_/Au junctions. (a) Schematic of the tunneling junction (left panel). False color scanning electron microscopy (SEM) image of the device (right panel). (b) Log-scale *I*_dc_ vs *V*_b_ curves for different ITO/Lu_2_O_3_/Au junctions with different *d* and *C*. The potential barrier diagrams with negative *V*_b_ and positive *V*_b_ are on the top. (c) The Fowler-Nordheim representation of the tunneling currents for different junctions. (d) EL spectra for different *V*_b_. The vertical bars indicate cutoff wavelengths based on *V*_b_. Inset shows the EL map of the *d* ≈ 2 nm Lu_2_O_3_ tunneling junction with *V*_b_ = −1.9 V. (e) Statistical data of the tunneling currents of the *d* ≈ 2 nm junction. Left axis: The maximum current fluctuations max­(δ*I*_dc_) as a function of *V*_b_ with regards to the average current in 5 days (purple shade). The current standard deviation σ­(*I*_dc_) as a function of *V*_b_ (green shade: 5 days; yellow shade: 1 h). Right axis: the relative standard deviation of *I*_dc_ (RSD), which is also called the coefficient of variation, of the currents as a function of *V*_b_ showing the variation of the currents. The dash lines indicate the low signal-to-noise area, where the maximum variation can approach 34% when *V*_b_ = 0 V, due to the limitation of the sourcemeter.

The current–voltage (*I*–*V*) characteristics, *I*_dc_ vs *V*_b_, of the ITO/Lu_2_O_3_/Au junctions with varying *d* and *C*, measured using a sourcemeter, reveal distinct tunneling characteristics (Figure 2b). |*I*_dc_| shows exponential growth with increasing *V*_b_ and, conversely, exponential decay with increasing *d*. For the *d* ≈ 6 nm Lu_2_O_3_ junction, *I*_dc_ does not exhibit clear tunneling behavior and remains in the picoampere range, approaching the limitation of our sourcemeters’ measurement capability. For the *d* ≈ 2 nm and *d* ≈ 3 nm junctions, the left branches of *I*_dc_ vs *V*_b_ (Figure 2b, *V*_b_ < 0 V) show higher currents compared to the right branches (*V*_b_ > 0 V), indicating the presence of asymmetric barriers within the junction due to dissimilar electrodes.

Furthermore, the Fowler-Nordheim representation, In­(*I*_dc_/*V*_b_^2^) vs *V*_b_^–1^, provides a clearer visualization of this asymmetry (Figure 2c). The left branches (*V*_b_ < 0 V) approach the turning point at lower voltages than the right branches (*V*_b_ > 0 V). Since the left turning point depends on the barrier ψ_2_ between Cr/Au and Lu_2_O_3_ and the right one depends on the barrier ψ_1_ between ITO and Lu_2_O_3_, this counterintuitively suggests that ψ_1_ is higher than ψ_2_, consistent with the fitting results via Simmons’s formula (Supporting Information, Section S3). It should be noted that the simple trapezoidal barrier diagram with the correction of image forces and Simmons’s formula are employed only to phenomenologically represent the tunneling process. In practice, besides work functions, several factors, such as interface roughness, defects, and oxygen vacancies, can also influence effective barrier profiles^48,54–56^ and lead to more complex asymmetric band diagrams. The simple model cannot fully reveal the underlying physical mechanisms contributing to the complicated band diagram associated with defect states, especially at higher bias voltages. Additionally, under high bias conditions (*V*_b_ > 1.5 V), EL spectra are observed for the *d* ≈ 2 nm junction (Figure 2d). The EL intensities, *I*_EL_, are expected to be directly proportional to the tunneling current, *I*_dc_, increasing with rising bias voltage *V*_b_. The cutoff wavelength, λ_cut_ = *hc*/*eV*_b_ (*c* is the speed of light in vacuum), of the EL spectra are determined by *V*_b_, as indicated by the short bars in Figure 2d. This suggests that the EL photons predominantly result from inelastic tunneling, while a small number of residual photons may result from other above-threshold emission processes.^25,26,57^ The electron-to-photon conversion efficiencies (Supporting Information, Section S12) observed in this study are relatively low, primarily due to the use of unoptimized structures and materials.

With our state-of-the-art fabrication process, the ITO/Lu_2_O_3_/Cr/Au junctions exhibit exceptional tunneling properties, characterized by minimal current fluctuations (δ*I*_dc_) and a consistent current density (*J*) across the tunneling cross-section. The inset in Figure 2d shows a uniform distribution of EL signal at *V*_b_ = −1.9 V, indicating a homogeneous *J* distribution. Moreover, the tunneling current (*I*_dc_) remains stable during long-term measurements. For example, one junction maintained a stable and consistent *I*_dc_ for over a month, with more than 6 h of total bias time (−1.6 V ≤ *V*_b_ ≤ 1.6 V), which is the longest duration we have tested. Additionally, these junctions function reliably even after being stored in a desiccator for over a year. To assess tunneling stability, we conducted *I*_dc_ vs *V*_b_ measurements for the *d* ≈ 2 nm junction over 5 days, covering *V*_b_ from −1.6 to 1.6 V in 148 cycles (half of which included femtosecond laser illumination), with a total duration of approximately 5 h. The statistical analysis of dark tunneling current fluctuations $\delta I_{dc}=I_{dc}-\overset{®}{I_{dc}\;}$ (excluding laser illumination), where $\overset{®}{I_{dc}\;}$ is the average tunneling current, is presented in Figure 2e. These maximal fluctuations, (max­(δ*I*_dc_)) and the standard deviation of *I*_dc_ (σ­(*I*_dc_)) are 2 to 3 orders of magnitude smaller than *I*_dc_ and can be nearly disregarded. Except for the region near *V*_b_ = 0 V (highlighted by the blue dashed lines in Figure 2e, showing a low signal-to-noise ratio), the variation in tunneling current, expressed as the Relative Standard Deviation (RSD), $RSD\left(I_{dc}\right)=\sigma \left(I_{dc}\right)/\overset{®}{I_{dc}\;}$ , is only ≈0.53%. Notably, the RSD­(*I*_dc_) can be even smaller in a shorter measurement period. For example, RSD­(*I*_dc_) ≈ 0.26% for 11 cycles measured over 1 h. Additional statistical data on the *d* ≈ 3 nm junction’s stability is shown in Supporting Information, Section S14. This level of stability in the tunneling current allows us to utilize DC measurements effectively to investigate small current changes resulting from nanoscale light-matter interactions.

### 2.2 Photoassisted Tunneling in ITO/Lu_2_O_3_/Au Junctions

To investigate the impact of laser excitation on tunneling current (*I*_dc_) in ITO/Lu_2_O_3_/Au junctions, we illuminate the junctions with femtosecond laser pulses focused using a 100× objective (numerical aperture, NA = 0.9), while continuously monitoring the tunneling currents using a sourcemeter. For example, the tunneling current of the *d* ≈ 3 nm Lu_2_O_3_ junction excited with a 600 nm laser shows a noticeable difference from the dark tunneling current (Figure 3a). The current difference between the laser-illuminated current and the dark current, denoted as Δ*I*_dc_ = *I*_laser_ – *I*_dc_, scales exponentially with *V*_b_ (Figure 3a, insert). It should be noted that Δ*I*_dc_ originates only from a small area of the junction illuminated with the laser. Similarly, Δ*I*_dc_ with comparable excitation wavelengths, λ_ex_, at 750 and 800 nm and respective incident powers, *P*_ex_ = 6.32 mW and 6.91 mW, exhibits exponential decay as *d* increases, with the left branches exceeding the right branches (Figure 3b). Additional results for *I*_laser_ excited with different λ_ex_ for different *d* are provided in Supporting Information, Section S2.

**Figure 3:**
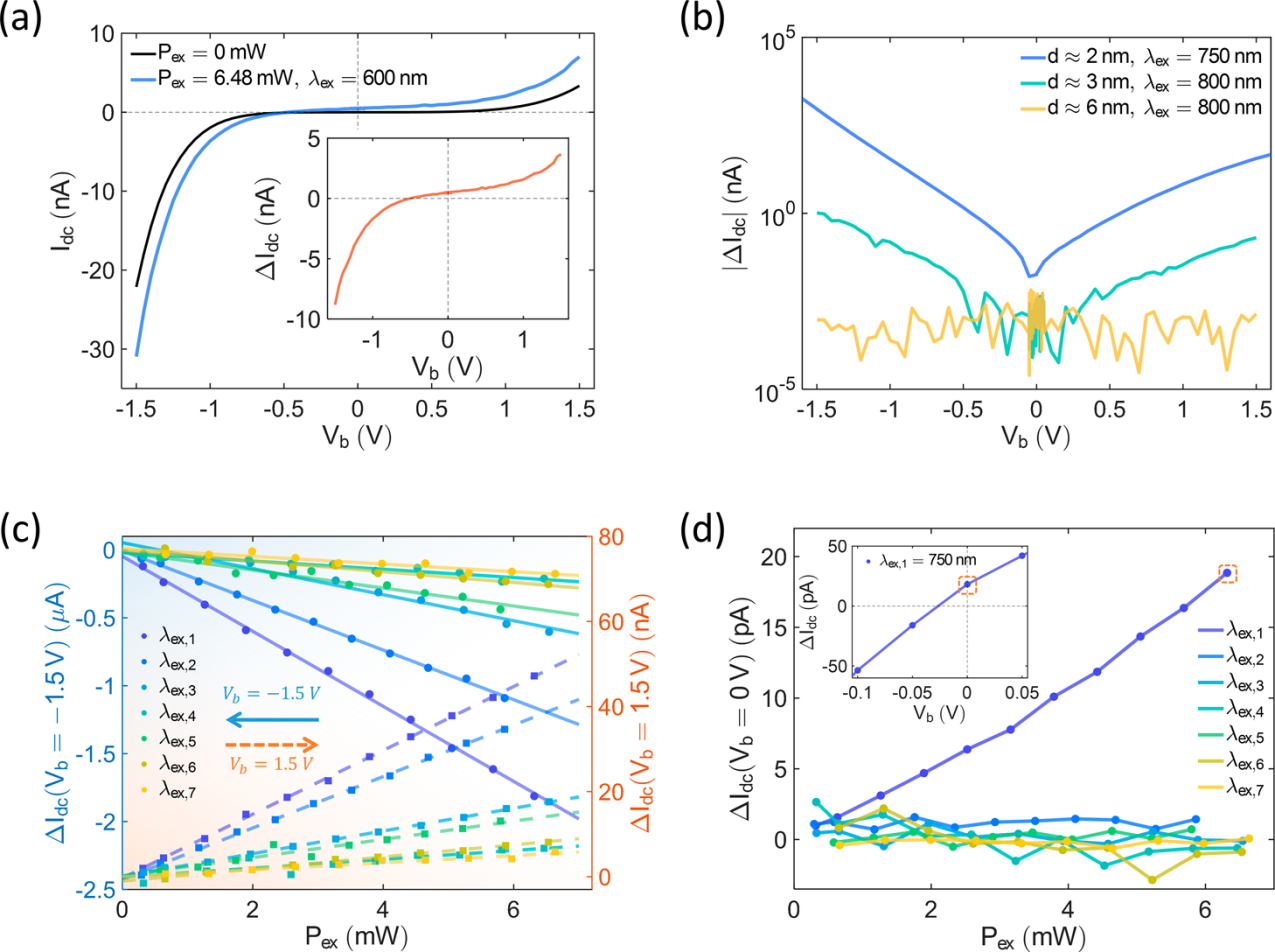
Laser-induced tunneling currents. (a) Tunneling current without laser illumination, *I*_dc_ (dark curve) and tunneling current with laser illumination, *I*_laser_ (blue curve) as a function of *V*_b_ in the *d* ≈ 3 nm Lu_2_O_3_ junction. The difference between them, Δ*I*_dc_, is shown in the inset. (b) Δ*I*_dc_ as a function of *V*_b_ for different tunneling junctions with different laser illumination. (c) Δ*I*_dc_ as a function of *P*_ex_ of different λ_ex,i_ in the 2 nm Lu_2_O_3_ junction, where λ_ex,*i*_=(650 + *i* × 100) nm. Circles and solid lines are the experimental data and the linear fitting curves for *V*_b_ = −1.5 V, respectively. Squares and the dash lines are the experimental data and the linear fitting curves with *V*_b_ = 1.5 V, respectively. (d) *I*_laser_ as a function of *P*_ex_ in the *d* ≈ 2 nm junction with different λ_ex,*i*_ at *V*_b_ = 0 V, where λ_ex,*i*_ = (650 + *i* × 100) nm. The inset shows Δ*I*_dc_ as a function of *V*_b_ at the small bias range with 6.32 mW, 750 nm laser illumination.

As mentioned above, various mechanisms can induce Δ*I*_dc_ in a tunneling junction, including thermal effects,^31,39–43^ hot carriers,^33–36^ optical rectification,^27–32^ and field emission current.^37,38^ Distinguishing these mechanisms is challenging but essential for developing a fundamental understanding and enabling practical device realization. To investigate the behavior of Δ*I*_dc_, we initially examined the *d* ≈ 2 nm junction under various λ_ex_ ranging from 750 to 1350 nm and different incident powers (*P*_ex_) (Figure 3c). The maximum Δ*I*_dc_ is achieved with λ_ex_ = 750 nm illumination at *P*_ex_ = 6.32 mW, constituting approximately 5% to 8% of *I*_dc_ across the range of *V*_b_ from −1.6 to 1.6 V. Notably, at *V*_b_ = −1.6 V, Δ*I*_dc_ reaches its largest value, Δ*I*_dc_(*V*_b_ = −1.6 V) = −(1.81 × 10^3^) nA. Since Δ*I*_dc_ exhibits the same sign as *V*_b_ for λ_ex_ values from 750 to 1350 nm, the possibility of a thermal voltage offset can be excluded. Additionally, thermal expansion cannot account for an increase in conductance within these junctions. From power-dependent measurements, it is evident that Δ*I*_dc_ is directly proportional to the incident power (*P*_ex_) for λ_ex_ ranging from 750 to 1350 nm (Figure 3c). These linear power dependences indicate that Δ*I*_dc_ is dominated by a one-photon process. This observation suggests that these current differences (Δ*I*_dc_) are not caused by thermal tunneling currents^41^ (more details are provided in Supporting Information, Section S4) or nonlinear field emission currents,^37,38^ which would exhibit nonlinear dependencies.

For the observed linear-power dependent Δ*I*_dc_, the most likely mechanisms are optical rectification or hot electron current due to internal photoemission process,^58,59^ as illustrated in Figure 1a,b, respectively. In the optical rectification process, the light-induced electric field modifies the potential barrier, thereby influencing current flow.^27^ Due to the asymmetry of the barrier, which can be influenced by factors such as bias voltage *V*_b_, electronic properties, and geometrical properties, the light-induced field can give rise to a net DC tunneling current—the optical rectification current, *I*_rect_. If the frequency of the incident light (ω_ex_) is sufficiently high, the energy of the electrons can surpass the barrier and generate current through photon absorption, a process known as hot-electron current.^33–35^

We proceed to measure the Δ*I*_dc_ (*V*_b_ = 0 V) of the *d* ≈ 2 nm Lu_2_O_3_ sample (Figure 3d). Except for λ_ex_ = 750 nm excitation, no nonzero Δ*I*_dc_(*V*_b_ = 0 V) is observed in Figure 3d. In the absence of bias voltage, both hot electrons and optical rectification can generate current at *V*_b_ = 0 V. The distinct boundary for λ_ex_ between nonzero and zero Δ*I*_dc_(*V*_b_ = 0 V) and the linear power-dependent Δ*I*_dc_(*V*_b_ = 0 V) suggest that the contribution comes from the hot-electron current (*I*_hot_). However, *I*_hot_ does not exhibit exponential growth with increasing *V*_b_, which results in a relatively weak hot-electron contribution to Δ*I*_dc_ at higher *V*_b_. Consequently, the optical rectification process is likely the dominant mechanism behind the observed laser-induced Δ*I*_dc_ in the *d* ≈ 2 nm sample when exposed to λ_ex_ ranging from 750 to 1350 nm, a topic that will be further discussed in the next section.

#### 2.2.1 Optical Rectification

First, we aim to determine the parameter range where optical rectification dominates the photoinduced electrical signal. DC currents generated through optical rectification when a tunneling junction is illuminated by a laser have been recently observed in various contexts, including nanogaps formed via electromigration,^28,31^ sandwich nanogaps,^60^ and molecular junctions.^29,30^ The theory of optical rectification is well-established and can be found in ref 27. For an excitation light with incident frequency ω_ex_ and light-induced voltage *V*_opt_ in the tunneling gap, the optical rectification current (*I*_rect_) can be approximated as^27–29^

2 $$I_{rect}=\frac{1}{4}V_{opt}^{2}\frac{\left[I_{dc}\left(V_{b}+\hbar \omega /e\right)+I_{dc}\left(V_{b}-\hbar \omega /e\right)-2I_{dc}\left(V_{b}\right)\right]}{{\left(\hbar \omega_{ex}/e\right)}^{2}}$$

where the second factor is the optical rectification factor, $\eta_{rect}=\left[I_{dc}\left(V_{b}+\hbar \omega /e\right)+I_{dc}\left(V_{b}-\hbar \omega /e\right)-2I_{dc}\left(V_{b}\right)\right]/{\left(\hbar \omega_{ex}/e\right)}^{2}$ , and *I*_dc_(*V*_b_) is the dark tunneling current with *V*_b_ bias. This equation shows that asymmetric potential barriers, mainly arising from the work function difference, play a critical role in optical rectification, particularly when *V*_b_ approaches 0 V (see Supporting Information, Section S11). If the conductance of the junction d*I*_dc_/d*V*_b_ varies slowly and the photon energy *ℏ*ω_ex_ is sufficiently low, the rectification factor can be approximated by the nonlinearity of the conductance, *G*_NL_ = (d^2^ *I*_dc_)/(d*V*_b_^2^). In this case eq 2 can be transformed into a classical description with a differential form.

3 $$I_{rect}=\frac{1}{4}V_{opt}^{2}\frac{d^{2}I_{dc}}{dV_{b}^{2}}$$

It is important to note that this equation does not account for any information regarding the incident light. In both eqs 2 and 3, the first factor 1/4*V*_opt_^2^ is proportional to both the incident power *P*_ex_, and the absorption of the junction for a specific wavelength *A*_ex_(λ_ex_). The second factor, involving *G*_NL_ and η_rect_, depends on the *I*–*V* characteristics of the junction. The challenge with using eq 2 is that measuring the dark current with a bias of (*V*_b_ + *ℏ*ω/*e*) may lead to junction breakdown and unstable currents due to the high bias, along with other limitations inherent to tunneling junctions. Therefore, despite its limitations, eq 3 remains widely used to investigate the optical rectification phenomenon. Based on these two equations, it is easy to predict the linear power dependence of *I*_rect_ for a fixed *V*_b_ and the exponentially growing *I*_rect_ with *V*_b_ for a fixed *P*_ex_, as observed in Figure 3b,c, respectively.

Furthermore, as indicated by eqs 2 and 3, for a fixed *P*_ex_ in the same junction, *I*_rect_ should be directly proportional to *G*_NL_ or η_rect_, because *I*_rect_ ∝ *A*_ex_ *P*_ex_ *G*_NL_ or *I*_rect_ ∝ *A*_ex_ *P*_ex_η_rect_, where *A*_ex_ is a constant for a specific λ_ex_ in the same junction. In Figure 4a, Δ*I*_dc_ of the *d* ≈ 2 nm Lu_2_O_3_ junction, excited with λ_ex_ = 750, 850, and 950 nm, indeed shows a clear linear dependence on *G*_NL_extracted from the dark tunneling currents *I*_dc_. The slopes of these linear relationships are determined by the 1/4*V*_opt_^2^ factor. Furthermore, as shown in the inset, the slopes of these linear relationships change at the 0 V turning point of *V*_b_. This phenomenon can be mainly explained by the difference between *G*_NL_ and η_rect_. Additionally, the inhomogeneous distribution of the light-induced field within the gap and the hot-electron effects may also contribute. Considering the slopes 1/4*V*_opt_^2^ extracted from the fitting and *G*_NL_ extracted from the dark current, the calculated optical rectification currents, 1/4*V*_opt_^2^ × *G*_NL_, match well with the measured Δ*I*_dc_ (see Supporting Information, Figure S5). Therefore, these current differences (Δ*I*_dc_) are dominated by the optical rectification process. Δ*I*_dc_ under CW-laser excitation exhibits similar behavior (see Supporting Information, Section S10).

**Figure 4:**
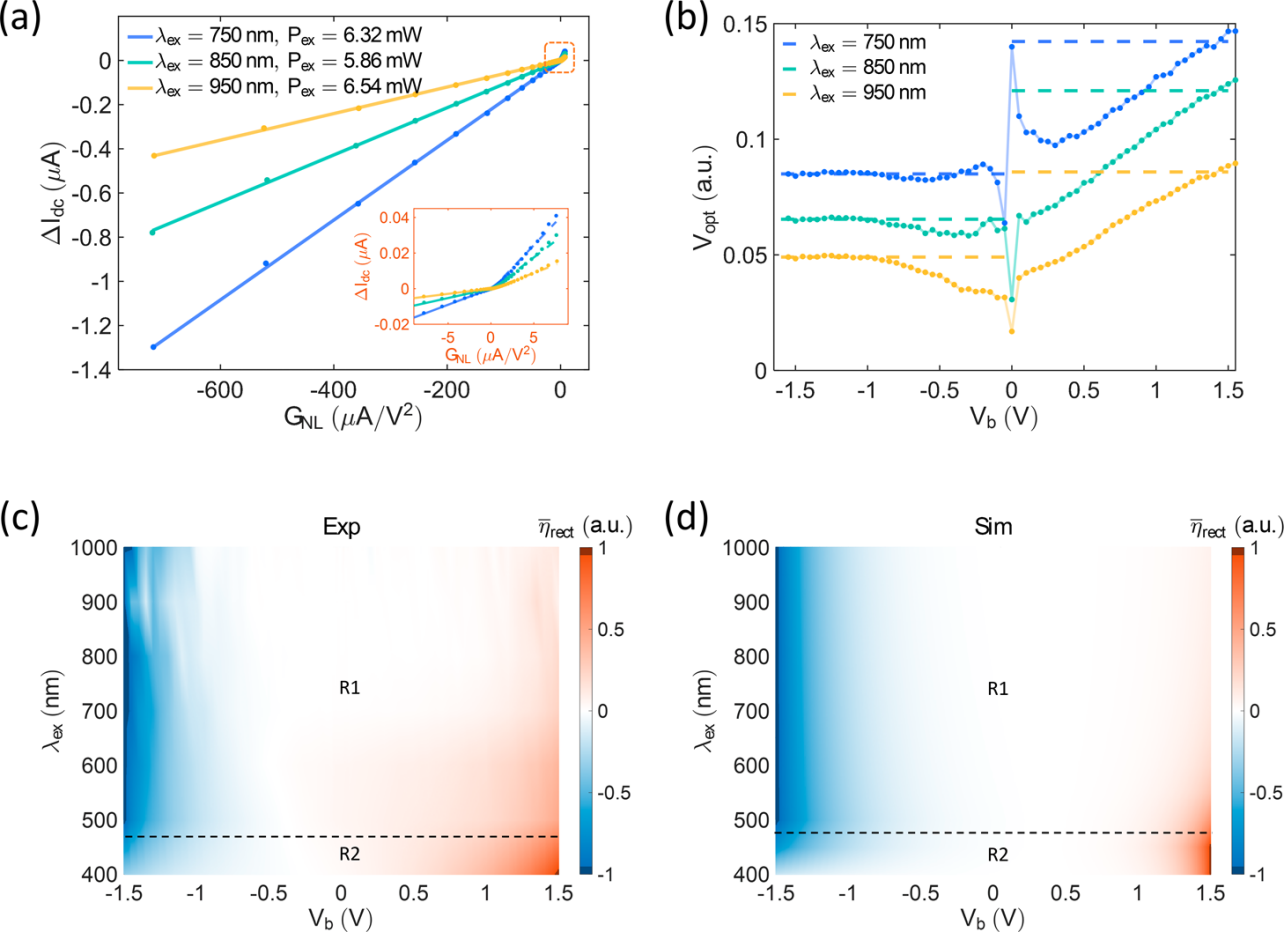
Optical rectification currents in tunneling junctions. (a,b) Experimental results based on the *d* ≈ 2 nm Lu_2_O_3_ junction. (a) Δ*I* as a function of the nonlinearity of the conductance, *G*_nl_, with different laser illuminations. Inset shows the zoomed-in area of the nonlinearity near 0. (b) Effective light-induced voltage *V*_op_ as a function of *V*_b_. Dots: *V*_op_ extracted from Δ*I*_dc_/(d^2^ *I*_dc_/d*V*_dc_^2^). Dashed lines: *V*_opt_ extracted from the fitting slopes in (a). (c) Map of ${\mathrm{\eta ̅}}_{rect}$ extracted by the fitting of Δ*I*_dc_ vs *P*_ex_ with the changes of the incident wavelength and the bias voltage, in the *d* ≈ 3 nm junction. (d) Map of ${\mathrm{\eta ̅}}_{rect}$ calculated by an equivalent asymmetric tunneling model.

The good agreement between the calculations based on eq 3 and the experimental data demonstrates the presence of optical rectification. However, eq 3, lacking information about the incident photons, can lead to differences from the results based on eq 2 or the experimental data, such as the unexpected turning points in Figure 4a. Due to the exponential nature of tunneling *I*–*V* characteristics, small currents might be overshadowed by larger ones during the fitting process. To clarify the limitations of eq 3, we calculate $V_{opt}={\left(4\Delta I_{dc}/G_{NL}\right)}^{1/2}$ as a function of *V*_b_ based on the experimental data under different excitations (Figure 4b). When compared to the *V*_opt_ (dashed lines in Figure 4b) extracted from the fitting slopes in Figure 4a, the left branches of the calculated *V*_opt_ with negative *V*_b_ show good agreement and remain relatively constant. However, their right branches with positive *V*_b_ deviate from the results obtained from the fitting slopes and display a continuous increase with *V*_b_. These deviations can be mainly attributed to the differences between *G*_NL_ and η_rect_ as *ℏ*ω_ex_ cannot be ignored. Therefore, *V*_opt_ calculated via eq 3 is not constant but varies with *V*_b_. Simultaneously, the scaled optical rectification factors match *G*_NL_ in the left branches (*V*_b_ < 0 V) but exhibit mismatches in the right branches (*V*_b_ > 0 V) (see Supporting Information, Figure S5), mirroring the findings of Figure 4c. As the photon energy *ℏ*ω_ex_ increases (or λ_ex_ decreases), the discrepancy between *G*_NL_ and η_rect_ becomes more pronounced. Based on eq 1, when the positive *V*_b_ is sufficiently strong to reach the Fowler-Nordheim tunneling region, the right branches of the tunneling currents can exceed the left branch due to the asymmetric barrier.^61^ According to the quantum description of eq 2, *I*_rect_ with a positive *V*_b_ can become larger than that with a negative *V*_b_, a result of the inverted rectification factor η_rect_ induced by the high photon energy—an effect that cannot be captured by the classical description of eq 3. This prediction is successfully observed in the *d* ≈ 3 nm junction with an excitation wavelength approaching 400 nm, where |Δ*I*_dc_(|*V*_b_|)|>|Δ*I*_dc_(−|*V*_b_|)| in the range −1.5 V ≤ *V*_b_ ≤ 1.5 V (see Supporting Information, Figure S2). The normalized optical rectification factor, ${\mathrm{\eta ̅}}_{rect}=\eta_{rect}\left(\lambda_{ex},V_{b}\right)/\max\left(|\eta_{rect}\left(\lambda_{ex},V_{b}\right)|\right)$ , where η_rect_(λ_ex_,*V*_b_) is calculated by fitting Δ*I*_dc_(*V*_b_) as a function of *P*_ex_, at positive *V*_b_ begins to surpass the values at negative *V*_b_ in region *R*2 shown in Figure 4c. It is a characteristic feature of quantum treatment—the deviation between η_rect_ and *G*_NL_— arising from the explicit involvement of photon energy in the rectification process. To validate this claim, an asymmetric trapezoid barrier tunneling model (see Supporting Information, Section S4) is used to simulate this behavior with eq 2 (quantum description) in Figure 4d. The result shows excellent agreement with the experimental data in Figure 4c, demonstrating that the substantial changes in Δ*I*_dc_ can be attributed to the optical rectification factor η_rect_.

#### 2.2.2 Hot Electrons and Thermal Effects

Beyond the optical rectification regime, we now address the parameter range where thermal and hot electron effects dominate. As discussed earlier, the Δ*I*_dc_ observed in the *d* ≈ 2 nm sample shows a linear dependence on *P*_ex_ and does not exhibit significant Δ*I*_dc_(*V*_b_ = 0 V) except under λ_ex_ = 750 nm excitation. Consequently, the thermal and hot electron effects can be disregarded. However, the influence of these effects becomes evident when the photon energy *ℏ*ω_ex_ is sufficiently high, and the incident power *P*_ex_ or absorption *A*_ex_ is strong enough.

The hot-electron current induced via one-photon internal photoemission, *I*_hot_, is linearly dependent on the incident power *P*_ex_ and influenced by incident photon frequency ω_ex_. A higher ω_ex_ provides electrons with more energy to overcome the barrier, allowing 0 V-bias currents, Δ*I*_dc_ (*V*_b_ = 0 V), to occur. As shown in Figure 5a, Δ*I*_dc_ (*V*_b_ = 0 V) is observed as λ_ex_ approaches 750 nm for the *d* ≈ 2 nm junction, 700 nm for the *d* ≈ 3 nm junction, and 600 nm for the *d* ≈ 6 nm junction. For the *d* ≈ 2 nm and the *d* ≈ 3 nm junctions, the cutoff wavelength λ_cut_ is around 700 nm, which can match the barrier height ψ ≈ 1.7 eV extracted from the Simmons’ equations (Supporting Information, Section S3). Δ*I*_dc_ (*V*_b_ = 0 V) at 700 nm excitation disappears in the *d* ≈ 6 nm junction sample, possibly due to the limitation of our sourcemeter. Δ*I*_dc_ (*V*_b_ ≠ 0 V) can be obviously observed with 700 nm excitation however it totally disappears with 800 nm excitation (Supporting Information, Figure S2), which not only indicates that hot electron currents dominate Δ*I*_dc_ but also shows direct evidence of the barrier height value. The observed cutoff behavior as a function of the excitation wavelength for Δ*I*_dc_ (*V*_b_ = 0 V), suggests that hot-electron currents, rather than thermal effects, are the primary mechanism driving Δ*I*_dc_ at *V*_b_ = 0 V. When the junctions with different widths *d* are excited respectively by λ_ex_ = 700 and 750 nm light, the linear power dependencies of Δ*I*_dc_ (*V*_b_ = 0 V) also align with the behavior of hot-electron currents. Moreover, according to eq 2, η_rect_ for both the *d* ≈ 2 and 3 nm junctions with λ_ex_ = 700 and 750 nm should be negative around *V*_b_ = 0 V, since |*I*_dc_(|*V*_b_|)| ≪ |*I*_dc_(−|*V*_b_|)| in the range −1.5 V ≤ *V*_b_ ≤ 1.5 V. Thus, the positive Δ*I*_dc_ (*V*_b_ = 0 V) can only be generated by hot electrons. As shown in Figure 4b, with λ_ex_ = 750 nm excitation, an abnormal peak in *V*_opt_ near *V*_b_ = 0 V is caused by the hot electron current *I*_hot_, as indicated by the nonzero Δ*I*_dc_(*V*_b_ = 0 V) shown in Figure 3d. This peak diminishes as *V*_b_ increases because optical rectification begins to dominate Δ*I*_dc_. The absence of nonzero Δ*I*_dc_(*V*_b_ = 0 V) with other λ_ex_ in Figure 3d due to ω_ex_ being too low to excite *I*_hot_, and η_rect_(*V*_b_ ≈ 0 V) being too small to generate detectable *I*_rect_. The contribution of hot electrons to Δ*I*_dc_ increases with *V*_b_, even when ω_ex_ cannot support nonzero Δ*I*_dc_(*V*_b_ = 0 V), as hot electrons can still tunnel through the barrier (tunneling of excited electrons). For λ_ex_ = 400 nm, the *G*_NL_ of the 3 nm Lu_2_O_3_ junction is used to calculate the effective optical voltages, *V*_opt_, which are much higher than expected in the small |*V*_b_| range (Supporting Information, Figure S6), indicating the contribution of hot-electron currents. In conclusion, while the overall trends of Δ*I*_dc_ remain primarily influenced by optical rectification at higher *V*_b_, due to the exponentially growing *I*_rect_, hot electron current (*I*_hot_) becomes the primary contributor to Δ*I*_dc_ in the |*V*_b_| range approaching 0 V, provided λ_ex_ is short enough.

**Figure 5:**
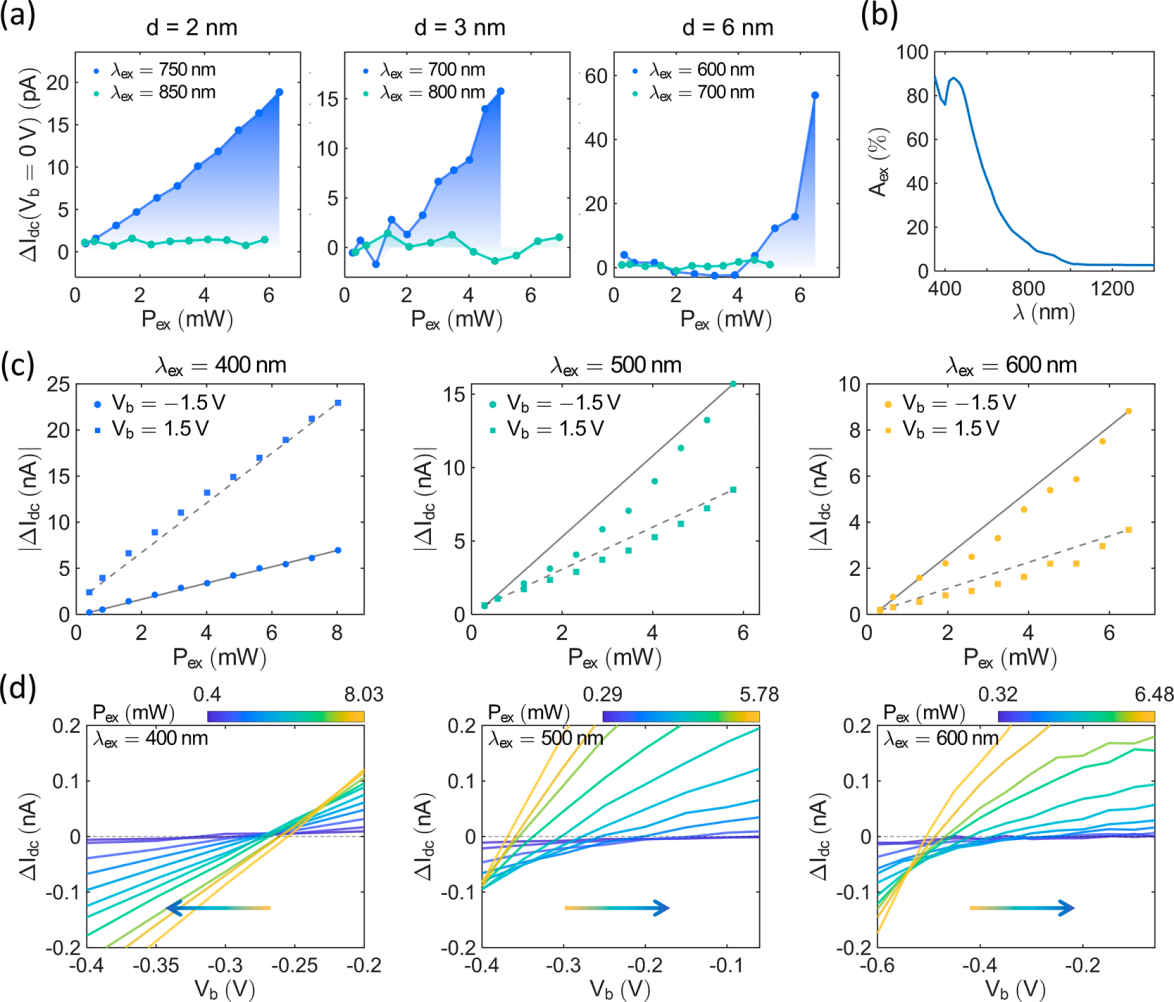
Tunneling currents affected by hot electrons and thermal effects. (a) 0 V-bias currents Δ*I*_dc_(*V*_b_ = 0 V) change with *P*_ex_ and λ_ex_ for different junctions, *d* ≈ 2 nm (left), *d* ≈ 3 nm (middle), and *d* ≈ 6 nm (right) respectively. (b) Simulated absorption spectrum of the *d* ≈ 3 nm junction. (c) For the *d* ≈ 3 nm junction, |Δ*I*_dc_| with *V*_b_ = −1.5 and 1.5 V biases as a function of *P*_ex_ for different λ_ex_, 400 nm (left), 500 nm (middle), and 600 nm (right). (d) Δ*I*_dc_ as a function of *V*_b_ near the open-circuit voltage range, for the*d* ≈ 3 nm junction are excited with λ_ex_ = 400 nm laser (left), λ_ex_ = 500 nm laser (middle), and λ_ex_ = 600 nm laser (right), respectively. The cross points between the solid lines and the dashed line indicate the open-circuit voltages, *V*_oc_. The arrows show the trend of *V*_oc_.

To understand the thermal effects, we simulate the absorption of the tunneling junction using rigorous coupled wave analysis (RCWA).^62^ The peak of the *d* ≈ 3 nm junction’s absorption ranges from 400 to 600 nm (Figure 5b) and it is expected that higher absorption levels contribute to thermal effects. Under λ_ex_ = 500 nm and 600 nm excitations, Δ*I*_dc_ at both −1.5 and 1.5 V biases display slight nonlinear dependence on *P*_ex_ (Figure 5c). Although multiphoton effects can lead to nonlinear power dependence, the power-law exponents in these cases remain close to 1, suggesting a low possibility of significant multiphoton contributions (see Supporting Information, Section S9). When the junction is illuminated at λ_ex_ = 400 nm, Δ*I*_dc_ show a slight nonlinear trend in the reverse direction. The thermal effects are related to the changes of the electron distributions due to the temperature, with several possible factors contributing to the nonlinearity of the curves. First, as discussed in ref 41, thermal tunneling currents, which increase with temperature, can lead to nonlinear power-dependent Δ*I*_dc_. An asymmetric trapezoid barrier tunneling model (see Supporting Information, Section S4) is used to simulate the thermal tunneling currents in the *d* ≈ 3 nm junction at different temperatures, showing that the thermal tunneling current increases with temperature (see Supporting Information, Section S7). Second, the optical rectification factor η_rect_ is expected to change with temperature due to variations in *I*_dc_, resulting in a nonlinear Δ*I*_dc_. Using the same tunneling model, the thermal optical rectification currents are simulated and found to increase with the temperature when *V*_b_ is away from 0 V (Supporting Information, Section S7). When *V*_b_ approaches 0 V, however, the trend reverses due to the specific η_rect_ resulting from the asymmetric barriers. Third, the thermal voltage caused by the temperature difference between two electrodes can also contribute to Δ*I*_dc_.^40^ Similarly, hot-electron currents can be affected by the temperature as well. Due to the overlapping conditions, thermal effects usually combine with hot-electron currents, creating a more complex Δ*I*_dc_. According to ref 34, the open-circuit voltage, ${V_{oc}=V}_{b}\left(\Delta I_{dc}=0\right)$ , caused by hot electrons should remain constant for the same λ_ex_ and be independent of *P*_ex_. If η_rect_ has a different sign from *I*_hot_, *I*_hot_ and *I*_rect_ can compete, resulting in changes in *V*_oc_. However, *V*_oc_ should still be independent of *P*_ex_ because both *I*_hot_ and *I*_rect_ exhibit linear power dependence. In the *d* ≈ 3 nm Lu_2_O_3_ junction, interestingly, *V*_oc_ with λ_ex_ = 400 nm, 500 nm, and 600 nm illuminations change with *P*_ex_, and the trends differ in directions (Figure 5d). These abnormal changes show nonlinear power-dependent processes, suggesting the involvement of competing thermal effects (discussed in Supporting Information, Section S7).

### 2.3 Photon-to-Current Responsivity

Previous sections have demonstrated that optical rectification primarily governs the current difference Δ*I*_dc_, while thermal effects and hot electrons contribute to Δ*I*_dc_ under specific conditions at small bias values. These combined contributions of Δ*I*_dc_ make the Lu_2_O_3_ tunneling junction a promising photodetector, capable of providing rich information about incident light, as Δ*I*_dc_ is sensitive to three factors: the incident power *P*_ex_, the photon wavelength λ_ex_, and the bias voltage *V*_b_. The junctions’ responsivities to incident light—the photon-to-current responsivity, η_pc_ = Δ*I*_dc_/*P*_ex_—vary with the thickness of the Lu_2_O_3_ layer *d* and the incident photon frequency ω_ex_ (Figure 6a–c). The *d* ≈ 2 nm Lu_2_O_3_ junction exhibits the highest η_pc_ with a responsivity at *V*_b_ = −1.5 V reaching up to 1.46 × 10^–7^ A/mW, which is considerably higher than at *V*_b_ = 1.5 V. Its η_pc_(ω_ex_) remains almost constant for different *P*_ex_, due to the dominance of optical rectification. In the *d* ≈ 3 nm Lu_2_O_3_ junction, the difference in η_pc_ at *V*_b_ = −1.5 V and *V*_b_ = 1.5 V is smaller than that of the *d* ≈ 2 nm junction, and η_pc_ at *V*_b_ = −1.5 V becomes even smaller than at *V*_b_ = 1.5 V when the photon energy *ℏ*ω_ex_ approaches ≈3 eV. When ω_ex_ > 2 eV, η_pc_ clearly changes with *P*_ex_ due to thermal effects. For both λ_ex_ = 500 and 600 nm, η_pc_ at both *V*_b_ = 1.5 V and −1.5 V increases with *P*_ex_. However, due to competing thermal currents, η_pc_ at *V*_b_ = 1.5 V decreases with *P*_ex_, while η_pc_ at *V*_b_ = −1.5 V remains constant under λ_ex_ = 400 nm illumination. As expected, the *d* ≈ 6 nm Lu_2_O_3_ junction exhibits the lowest η_pc_, with η_pc_ at *V*_b_ = 1.5 V being higher than that at *V*_b_ = −1.5 V. For the *d* ≈ 2 nm and *d* ≈ 3 nm Lu_2_O_3_ junctions, these changes in η_pc_ are mainly attributed to variations in η_rect_, due to the asymmetric barrier and the absorption characteristics of the tunneling junction. The impact of the asymmetric barrier on η_pc_ is discussed in Supporting Information, Section S8. Furthermore, η_pc_ is expected to increase exponentially with *V*_b_ due to the intrinsic tunneling properties. For example, when *V*_b_ increases to 1.6 V, η_pc_ of the *d* ≈ 2 nm junction under λ_ex_ = 750 nm can reach up to 2.89 × 10^–7^A/mW, corresponding to the photon-to-electron conversion efficiency of approximately 4.78 × 10^–4^ and the optical power-to-electrical power conversion efficiency of approximately 4.62 × 10^–4^. Additional data on photon-to-electron conversion efficiencies and optical power-to-electrical power conversion efficiencies are provided in Supporting Information, Section S13. In the *d* ≈ 6 nm Lu_2_O_3_ junction, it should be noted that hot electrons or thermal effects may dominate its η_pc_, due to its weak η_rect_. In conclusion, the tunable asymmetric potential barriers and the engineerable structure of the Lu_2_O_3_ junctions offer multiple mechanisms—including optical rectification, hot electron, and thermal effects—making them sensitive detectors for studying light–matter interactions at the nanoscale.

**Figure 6:**
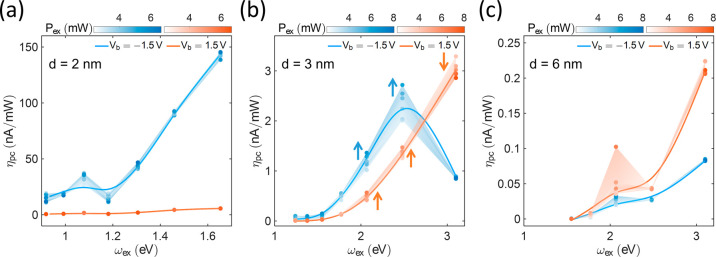
Photon-to-current responsivity. η_pc_ for different excitation powers *P*_ex_ of the *d* ≈ 2 nm (a), *d* ≈ 3 nm (b), and *d* ≈ 6 nm (c) junctions, biased at −1.5 V­(blue) and 1.5 V (red).

## 3 Conclusions

In summary, the ultrastable epitaxial Lu_2_O_3_ tunneling junctions realized in this work offer stable tunneling current enabling long-term operation while allowing for reliable extraction of *I*–*V* characteristics, including sensitive light-induced Δ*I*_dc_ and nonlinear conductance *G*_NL_ measurements. The photoassisted tunneling phenomena observed in these tunneling junctions are attributed to three mechanisms: optical rectification, hot-electron current, and thermal tunneling current. By analyzing changes in Δ*I*_dc_ under different experimental conditions, we conclude that optical rectification is the dominant factor in Δ*I*_dc_, especially when the bias voltage, *V*_b_, is high. In the spectral range with high absorption, thermal tunneling contributions become evident, exhibiting a nonlinear dependence on input power. When the photon energy is sufficiently high, hot-electron current is shown to dominate Δ*I*_dc_ at small *V*_b_. Simultaneously, the results provide direct experimental evidence of the effects of photon energy in optical rectification and the deviations between classical and quantum descriptions of optical rectification in tunnel junctions.

Additionally, the photon-to-current efficiency realized here is determined by the geometry of the planar sandwich junctions. However, nanostructuring the top Au electrode to excite plasmon resonances could allow tuning of the absorption spectrum, enhancing photon-to-current efficiency, or altering the hot-electron generation rate. The tunneling barrier can also be designed with increased asymmetry to amplify the optical rectification process. Given these advantages, therefore, the ultrastable Lu_2_O_3_ tunneling junctions realized here serve as a promising platform for a range of nanophotonic applications, including both light emission and detection.

## 4 Methods

### 4.1 Sample Fabrication

The ITO bottom electrode is epitaxially grown on an yttria-stabilized zirconia (YSZ) substrate via pulsed laser deposition (PLD). Subsequently, a Lu_2_O_3_ layer of thickness *d* is epitaxially grown on top of ITO, again using PLD. Following this, a 2 nm-thick layer of Cr is deposited on the Lu_2_O_3_ as an adhesion layer, followed by a deposition of the top Au electrode of nominal thickness 50 nm via thermal evaporation. To isolate the Au electrode, a 200 nm amorphous aluminum oxide (AlO_*x*_) layer is deposited via PLD on top of the Lu_2_O_3_, except in the tunneling region. The detailed procedure can be found in ref 50.

### 4.2 Electrical Measurements

The current–voltage (*I*–*V*) characteristics of the tunneling junctions are measured via the sourcemeter (Keithley 2000). The measurements are controlled by a homemade program based on Matlab. For all experiments, the sourcemeter simultaneously applies the bias voltages and measures the currents in the junction, Supporting Information, Figure S1. The number of power line cycles (NPLC) is set to 10 for higher accuracy.

### 4.3 Electroluminescence and Laser Illumination Experiments

An inverted microscope (Olympus IX73) is used to conduct optical measurements of the tunneling junctions for both electroluminescence (EL) and the laser excitation experiments. For the EL spectrum measurements, the emitted photons are collected by a 100× objective (numerical aperture, NA = 0.9) and detected by a spectrometer (Andor Shamrock 303i) with a CCD (Andor iDUs), Supporting Information, Figure S1a. The sourcemeter is used to apply bias voltages and monitor the currents. For the laser illumination measurements, a femtosecond laser (∼150 fs, 80 MHz) emission is attenuated by neutral density (ND) filters and focused on the junctions using the 100× objective (NA = 0.9), Supporting Information, Figure S1b.

## Supplementary Material



## Data Availability

All data are available in the main text or the Supporting Information.

## Notes

Schematic diagram of experimental setups; featured *I*–*V* characteristics with laser illumination; tunneling *I*–*V* characteristic fitting model; equivalent tunneling model; optical rectification effects in the *d* ≈ 2 nm Lu_2_O_3_ junction; hot electron’s strong contribution to *V*_opt_; thermal effects; normalized photon-to-current responsivity; power dependence of the laser-induced tunneling current; laser-induced tunneling current under CW-laser excitation; effects of asymmetric barriers on optical rectification; electroluminescence (EL) in tunnel junction; conversion efficiency; stability of tunnel junctions; characterization of the epitaxially grown Lu_2_O_3_ junction (PDF)
