# Supplementary material for: Light-Matter Interaction in Ultrastable Tunneling Nanogaps
Source: ACS Nano. 2025 Jul 23;19(30):27204–14. doi: 10.1021/acsnano.5c03217 (PMC12333406; doi:10.1021/acsnano.5c03217)
Supplement: Supplementary file 1 [file nn5c03217_si_001.pdf]

Supporting Information for:

Light-matter interaction in ultra-stable tunneling nanogaps

Yuankai Tang[1], Saurav Prakash[2], Proloy Nandi[2], Ariando Ariando[2], Amit Agrawal[3,4], Hayk Harutyunyan[1]**

[1]Department of Physics, Emory University, Atlanta, GA 30322, USA.

[2]Department of Physics, National University of Singapore, Singapore 117551.

[3]Department of Engineering, University of Cambridge, Cambridge CB3 0FA, UK.

[4]Kyunghee University, 26 Kyungheedae-ro, Dongdaemun-gu, Seoul 02447, Korea

*Correspondence to: aka59@cam.ac.uk and hharuty@emory.edu

S1. Schematic Diagram of Experimental Setups

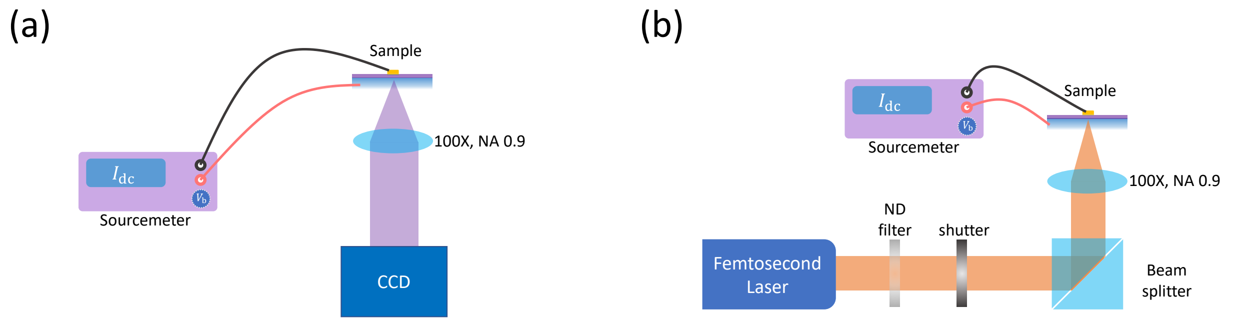

Figure S1. (a) Sketch of the EL measurements. (b) Sketch of the experiments with a laser illumination.

S2. Featured I-V Characteristics with Laser Illumination

The current differences between the laser-illuminated current and the dark current, $\Delta I_{dc} = I_{laser} - I_{dc}$, show clear trends with the Lu_2O_3 thicknesses d, the incident wavelengths λ_{ex}, and the incident powers P_{ex}. The thinner d results in a higher ΔI_{dc}. For all junctions, ΔI_{dc} increases with P_{ex} for the same λ_{ex}. For the $d \approx 6$ nm sample, there is no clear ΔI_{dc} when $\lambda_{ex} = 800$ nm, but ΔI_{dc} is observed when $\lambda_{ex} < 800$ nm. Thus, hot-electron current makes obvious contributions to ΔI_{dc}.

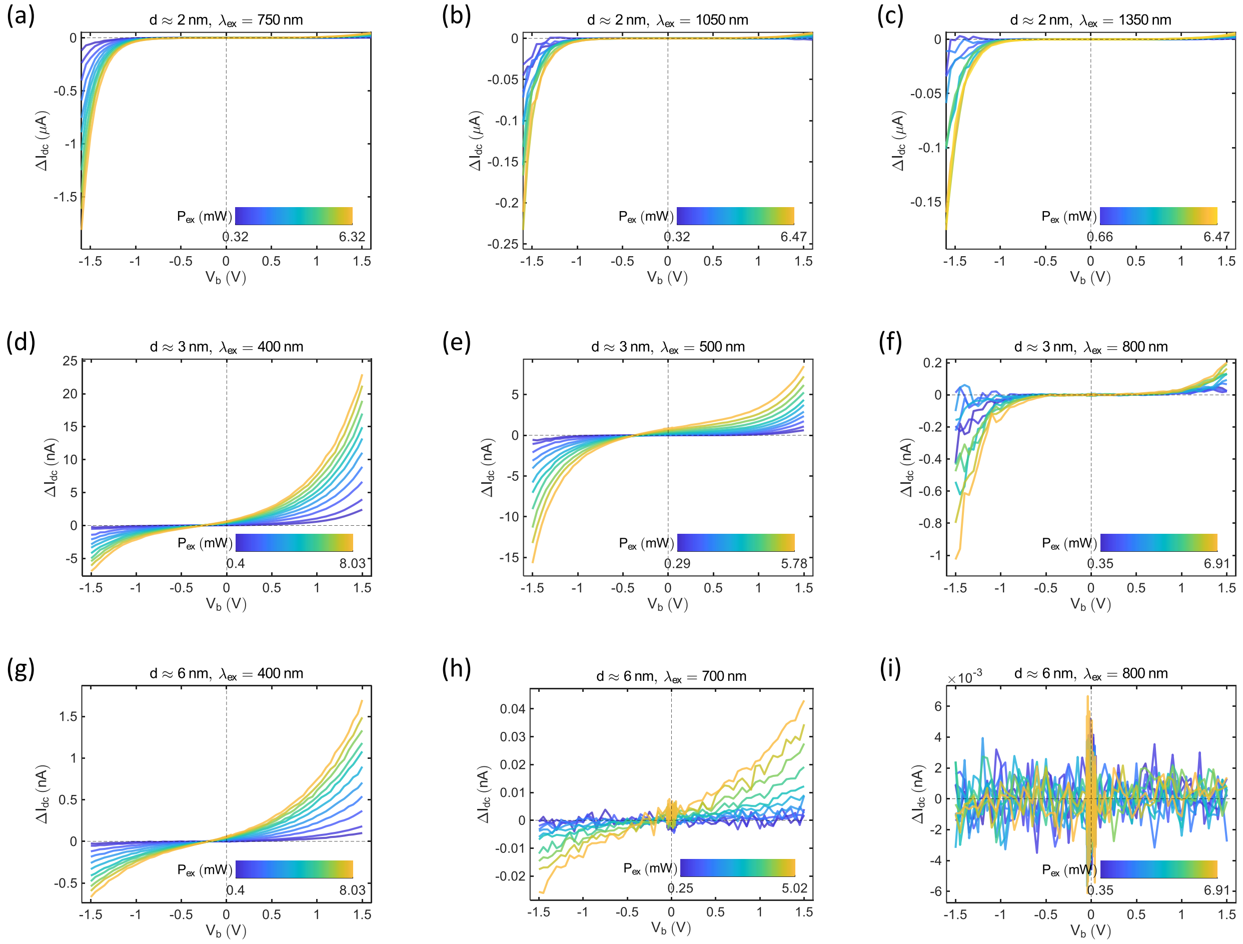

Figure S2. The tunneling current difference ΔI_{dc} as a function of V_{b} for three junctions with different Lu_2O_3 thickness d and laser wavelength λ_{ex} at various powers P_{ex}. (a-c) $d \approx 2$ nm, $\lambda_{\mathrm{ex}} = 750$ nm, 1050 nm, and 1350 nm. (d-f) $d \approx 3$ nm, $\lambda_{\mathrm{ex}} = 400$ nm, 500 nm, and 800 nm. (g-i) $d \approx 6$ nm, $\lambda_{\mathrm{ex}} = 400$ nm, 700 nm, and 800 nm.

S3. Tunneling I-V Characteristic Fitting Model

The tunneling I-V Characteristics can reveal information about the junctions, including the insulating layer thickness d and the potential barriers ψ. For extracting these parameters, a reasonable fitting model is necessary. Based on the WKB method, Simmons' equations give an approximation of the tunneling currents with a trapezoidal barrier, [1-3]

$$I_{\mathrm{dc}} = C \times \frac{e}{2\pi h(\beta\Delta s)^2} \times \left[\varphi \mathrm{e}^{-A\sqrt{\varphi}} - (\varphi + eV_{\mathrm{b}})\mathrm{e}^{-A\sqrt{\varphi+eV}}\right] \times J_T. \qquad (S1)$$

Here, C is the tunneling cross-sectional area, e is the charge of electron, h is the Planck's constant, and $A = 4\pi\beta\Delta s/h\sqrt{2m}$. $\Delta s = s_1 - s_2$ is the barrier width along the x-axis (from s_1 to s_2), where s_1 is the tunneling start point, ands_2 is the tunneling ending point (the area between s_1 and s_2 is the non-classical region), and it depends on the insulator thickness d, the bias voltage V_{b}, and $\psi(x) = \psi(0) - (\psi(0) - \psi(d) + eV_{\mathrm{b}}) \times x/d$ Considering the image potential $V_{\mathrm{im}}(x) \approx -1.15(e^2\mathrm{In}2)d/8\pi\varepsilon_r\varepsilon_0 x(d-x)$ due to the image force, $\psi_{\mathrm{im}}(x) = \psi(0) - (\psi(0) - \psi(d) + eV_{\mathrm{b}}) \times x/d + V_{\mathrm{im}}(x)$, where ε_r and ε_0 are the relative permittivity of the insulating material and the vacuum permittivity, respectively. The average potential barrier height becomes $\varphi = \int_{s_1}^{s_2} \psi_{\mathrm{im}}(x)dx/\Delta s$. $\beta = 1 - (1/(8\Delta s\varphi^2))\int_{s_1}^{s_2}(\psi_{\mathrm{im}}(x) - \varphi)^2 dx/\Delta s$ is the correction factor as a result of the approximation. β approaches 1 in many cases, [1, 2] however, β needs to be calculated accurately in our fitting model because it is the only contribution to the asymmetric properties of the junctions in the intermediate V_{b} range. [2, 4] The last factor is the temperature dependent factor, $J_T = (\pi kTA/2\sqrt{\varphi})/\sin(\pi kTA/2\sqrt{\varphi})$, where k is the Boltzmann's constant and T is the temperature.

Because of the complexity of the real potential barrier in the tunneling junctions, it is challenging to use the ideal trapezoidal barrier to describe the tunneling behavior for the large V_{b}. To estimate the asymmetric barrier heights ψ and the insulation thickness d, the tunneling I-V characteristics are fitted in the small bias range $-0.5\ \mathrm{V} < V_{\mathrm{b}} < 0.5\ \mathrm{V}$, and the temperature is set to $T = 300$ K for all the junctions without laser illumination, Figure S3. The fitting results agree with the designed parameters well. For both the $d \approx 2$ nm and 3 nm samples, the barrier ψ_1 between ITO and Lu_2O_3 is higher than the barrier ψ_2 between Lu_2O_3 and Cr/Au, and the ψ_1 is around 1.7 eV,

which indicates the cut-off wavelength of 0 V-bias hot-electron current should be around 729 nm. This conclusion agrees well with the results of the $\Delta I_{\mathrm{dc}}(V_{\mathrm{b}} = 0\ \mathrm{V})$ measurements.

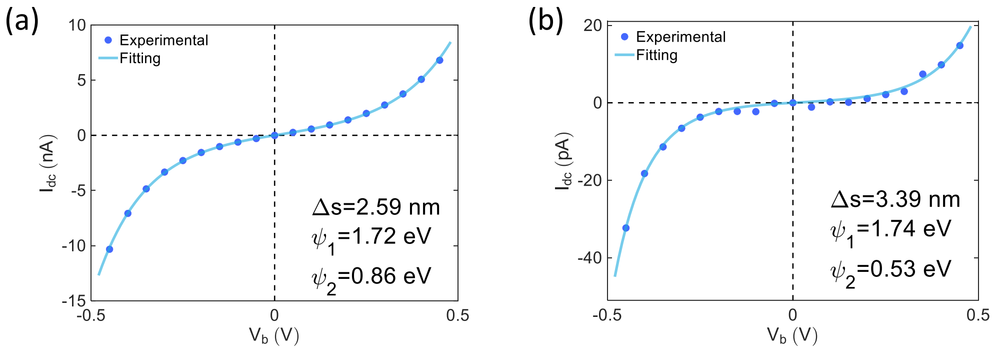

Figure S3. The fitting results of the tunneling characteristic based on the Simmons' equations. (a) I_{dc} as a function of V_{b} for the $d \approx 2$ nm junction. (b) I_{dc} as a function of V_{b} for the $d \approx 3$ nm junction. The dots are the experimental data, and the lines are the fitting curves.

S4. Equivalent Tunneling Model

As mentioned above, the ideal trapezoidal barrier model is unsuitable for the tunneling characteristics with large V_{b}. For our tunneling junctions, the potential barriers of ITO/Lu_2O_3 and Lu_2O_3/Cr/Au are complex, which cannot be treated as simple trapezoidal barriers and be extracted directly via the Simmons' equations, especially when V_{b} approaches ± 1.5 V. When the range of V_{b} expands to ± 1.5 V, for example, the parameters d, ψ_1, and ψ_2 of the $d \approx 3$ nm junction extracted from the fitting model are very different from the previous results for $-0.5\ \mathrm{V} < V_{\mathrm{b}} < 0.5\ \mathrm{V}$ (Figure S3b), as shown in Figure S4a. These parameters with $-1.5\ \mathrm{V} \leq V_{\mathrm{b}} \leq 1.5\ \mathrm{V}$ do not show a very good match with experimental results. Additionally, it should be noted that the fitting curve does not match with the experimental data well for small V_{b} in Figure S4a.

However, the fitting model can still be used as a reasonable equivalent model to estimate the tunneling behaviors for large V_{b}. For instance, based on the fitting parameters in Figure S4a, the

simulated normalized optical rectification factor $\bar{\eta}_{\mathrm{rect}}$ has a good agreement with the experimental data, as shown in Figure 4d. Here, we try to use the equivalent model with the fitting parameters in Figure S4a to examine whether $\Delta I_{\mathrm{dc}}(\lambda_{\mathrm{ex}} = 400\ \mathrm{nm})$ of the $d \approx 3$ nm junction can be just a result of the thermal effect. When $T = 965$ K, the thermal tunneling current can approach the tunneling current with the laser illumination, $I_{\mathrm{dc}}(T = 965\ \mathrm{K},\ V_{\mathrm{b}} = -1.5\ \mathrm{V}) \approx I_{\mathrm{laser}}(\lambda_{\mathrm{ex}} = 400\ \mathrm{nm}, P_{\mathrm{ex}} = 8\ \mathrm{mW})$, Figure S4b. However, their right branches (Figure S4b) are far away from each other, which indicates the thermal tunneling current is not the origin of $\Delta I_{\mathrm{dc}}(\lambda_{\mathrm{ex}} = 400\ \mathrm{nm})$. Simultaneously, with the assumption that the change of T is proportional to P_{ex} in the junction, the $\Delta I_{\mathrm{dc}}(T) = I_{\mathrm{dc}}(T) - I_{\mathrm{dc}}(T = 300\ \mathrm{K})$ is not linearly dependent on T which is different from the linear power dependent $\Delta I_{\mathrm{dc}}(P)$, Figure S4c.

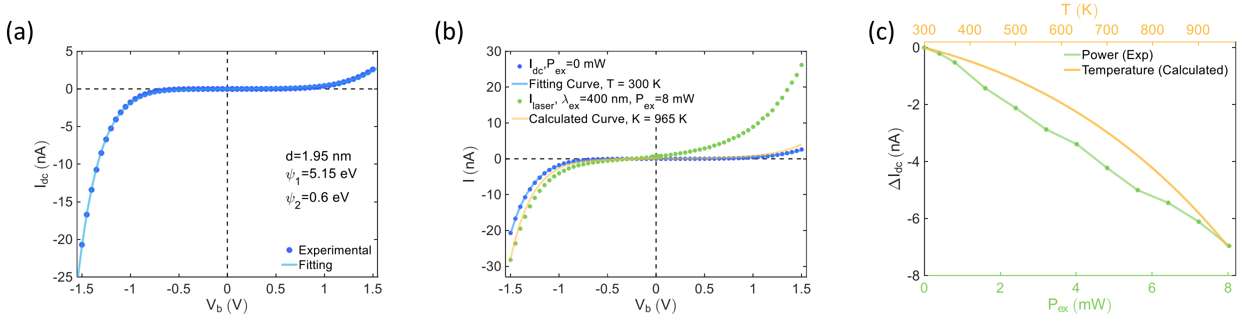

Figure S4. (a) I_{dc} as a function of V_{b} for the $d \approx 3$ nm junction. The dots are the experimental data, and the line is the fitting curve. (b) The blue dots and the light blue line are the experimental data and the fitting curve of the $I_{\mathrm{dc}}(T = 300\ \mathrm{K})$, respectively. The green dots are the experimental data of $I_{\mathrm{laser}}(\lambda_{\mathrm{ex}} = 400\ \mathrm{nm}, P_{\mathrm{ex}} = 8\ \mathrm{mW})$ as a function of V_{b}. The yellow curve is the calculated $I_{\mathrm{dc}}(T = 965\ \mathrm{K})$ as a function of V_{b}. (c) ΔI_{dc} as a function of P_{ex} (green dot and line, experimental data). ΔI_{dc} as a function of T (yellow line, calculated result).

S5. Optical Rectification Effects in the $d \approx 2$ nm Lu_2O_3 Junction

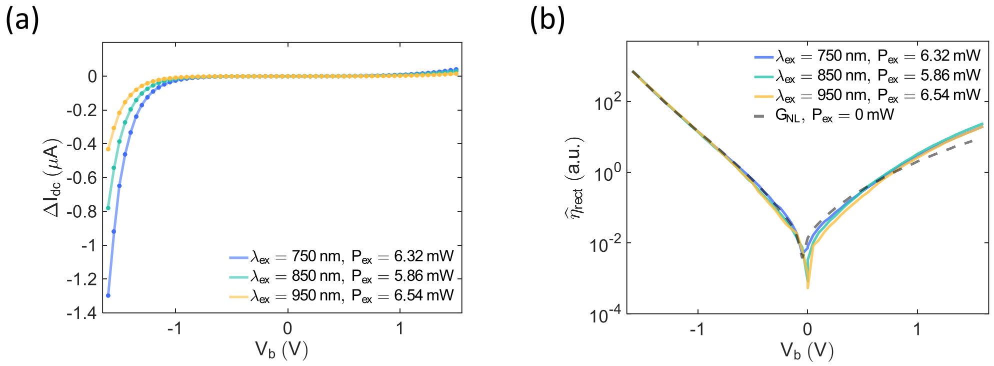

Figure S5. The results based on the $d \approx 2$ nm Lu_2O_3 junction. (a) The slopes $(1/4V_{\mathrm{opt}}^2)$ extracted form Figure 4a and the nonlinearity G_{nl} extracted form the dark tunneling current are used to calculate the ΔI_{dc} as a function of V_{b}. Dots: the expermental data. Lines: the calculated curves. The R^2 values for these matches are all greater than 0.99. (b) The gray dash line is $|G_{\mathrm{nl}}|$ exctracted form the dark tunneling current. The solid color lines are the scaled optical rectification factors $\hat{\eta}_{\mathrm{rect}} = |\eta_{\mathrm{rect}}(\lambda_{\mathrm{ex}}, V_{\mathrm{b}})|/|(\sum_{V_{\mathrm{b}}} \eta_{\mathrm{rect}}(\lambda_{\mathrm{ex}}, V_{\mathrm{b}}) \,/\, \sum_{V_{\mathrm{b}}} G_{\mathrm{nl}}(\lambda_{\mathrm{ex}}, V_{\mathrm{b}}))|$, where $\eta_{\mathrm{rect}}(\lambda_{\mathrm{ex}}, V_{\mathrm{b}})$ extracted by the fitting of ΔI_{dc} vs P_{ex}.

S6. Hot Electron's Strong Contribution to V_{opt}

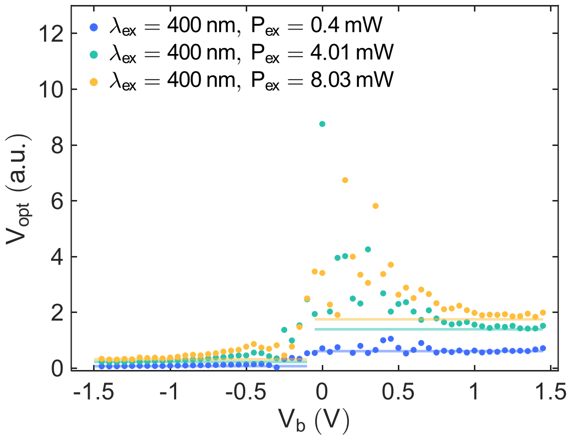

Figure S6. $V_{\rm opt}$ as a function of $V_{\rm b}$ for the $d = 3$ nm junction with $\lambda_{\rm ex} = 400$ nm. Dots are $V_{\rm opt}$ extracted from $\Delta I_{\rm dc}/(d^2 I_{\rm dc}/dV_{\rm b}^2)$ and the dash lines are $V_{\rm op}$ extracted from the fitting slopes. $V_{\rm opt}$ from the classical description offers an insight into the contribution of hot electrons. The peaks around $-0.2\ \mathrm{V} < V_{\rm b} < 0.5\ \mathrm{V}$ are caused by hot-electron currents. Although the substantial difference between $G_{\rm NL}$ and $\eta_{\rm rect}$ can also result in a mismatch, the trend of the effective $V_{\rm opt}$ caused by this difference will be reversed and increase with $|V_{\rm b}|$ (Figure 4b). Here, however, the effective $V_{\rm opt}$ decreases with $|V_{\rm b}|$ because it is boosted by the hot electrons at small $|V_{\rm b}|$. As $|V_{\rm b}|$ increases, the effective $V_{\rm opt}$ becomes smaller and more consistent, indicating that optical rectification once again dominates $\Delta I_{\rm dc}$.

S7. Thermal Effects

In the $d \approx 3$ nm junction, the changing open-circuit voltages, $V_{\rm oc} = V_{\rm b}(\Delta I_{\rm dc} = 0)$, are observed due to the thermal effects. The different trends with different $\lambda_{\rm ex}$ indicate the competing thermal contributions. These thermal contributions are all caused by the electron distributions in the two electrodes. There are four possible factors: the normal thermal tunneling current, $I_{\rm tun}^{\rm T}$, the thermal optical rectification current, $I_{\rm rect}^{\rm T}$, the thermalized "hot electron" current, $I_{\rm hot}^{\rm T}$, and the thermal current caused by the temperature difference between ITO and Au electrodes, $I_{\rm TD}^{\rm T}$. Here, the contribution of $I_{\rm TD}^{\rm T}$ is similar to $I_{\rm hot}^{\rm T}$ because of their similar mechanism. [5] In the region between 0 V and $V_{\rm oc}$, these four thermal currents can have different directions. $I_{\rm rect}^{\rm T}$, has the same sign as the negative $V_{\rm b}$ and flows from the Au electrode to the ITO electrode, resulting in a lower net current. The thermalized "hot electron" current, $I_{\rm hot}^{\rm T}$ and $I_{\rm TD}^{\rm T}$ flows from the ITO electrode to the Au

electrode (red arrow) due to the high absorption in Au electrode, resulting in a higher net current. The direction of the thermal optical rectification current, $I_{\mathrm{rect}}^{\mathrm{T}}$, depends on η_{rect}.

With $\lambda_{\mathrm{ex}} = 500$ nm ($\hbar\omega_{\mathrm{ex}} = 2.48$ eV) illuminations, $|\Delta I_{\mathrm{dc}}(V_{\mathrm{b}} = -1.5\ \mathrm{V})| > |\Delta I_{\mathrm{dc}}(V_{\mathrm{b}} = 1.5\ \mathrm{V})|$, as shown in Figure S2e, which implies $|I_{\mathrm{rect}}(V_{\mathrm{b}} = -1.5\ \mathrm{V})| > |I_{\mathrm{rect}}(V_{\mathrm{b}} = 1.5\ \mathrm{V})|$ if the contribution from I_{hot} is negligible. Because $|I_{\mathrm{dc}}(-3.98\ \mathrm{V})| \gg |I_{\mathrm{dc}}(-1.5\ \mathrm{V})|$and $|I_{\mathrm{dc}}(0.98\ \mathrm{V})|$, and $|I_{\mathrm{dc}}(3.98\ \mathrm{V})| \gg |I_{\mathrm{dc}}(1.5\ \mathrm{V})|$ and $|I_{\mathrm{dc}}(-0.98\ \mathrm{V})|$, $|I_{\mathrm{dc}}(-3.98\ \mathrm{V})| > |I_{\mathrm{dc}}(3.98\ \mathrm{V})|$ can be derived from Eq. (2). Thus, for both $\lambda_{\mathrm{ex}} = 400$ nm, $\lambda_{\mathrm{ex}} = 500$ nm, and $\lambda_{\mathrm{ex}} = 600$ nm illuminations, $|I_{\mathrm{dc}}(-\hbar\omega_{\mathrm{ex}}/e + V_{\mathrm{b}})| > |I_{\mathrm{dc}}(\hbar\omega_{\mathrm{ex}}/e + V_{\mathrm{b}}) - 2I_{\mathrm{dc}}(V_{\mathrm{b}})|$ is reasonable if V_{b} is in the range from 0 to V_{oc}. As a result, $I_{\mathrm{rect}}^{\mathrm{T}}$ has the same sign as $I_{\mathrm{tun}}^{\mathrm{T}}$ due to the negative η_{rect} in this bias range. Therefore, decreasing $|V_{\mathrm{oc}}|$ with P_{ex} from $\lambda_{\mathrm{ex}} = 400$ nm illumination is mainly caused by the sum of $I_{\mathrm{rect}}^{\mathrm{T}}$ and $I_{\mathrm{tun}}^{\mathrm{T}}$ (Figure 5d, left panel). However, $|V_{\mathrm{oc}}|$ with $\lambda_{\mathrm{ex}} = 500$ nm and 600 nm illumination has a opposite trend and increases with P_{ex} (Figure 5d, middle and right panels). In this case, $I_{\mathrm{hot}}^{\mathrm{T}}$ and $I_{\mathrm{TD}}^{\mathrm{T}}$ are strong enough to overcome the sum of $I_{\mathrm{rect}}^{\mathrm{T}}$ and $I_{\mathrm{tun}}^{\mathrm{T}}$. The directions of thermal currents are shown in Figure S7.

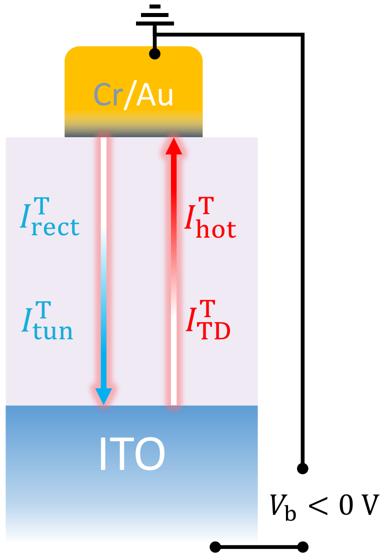

Figure S7. Sketch of the directions of thermal currents in the $d \approx 3$ nm junction with V_{b} near V_{oc}.

We apply the Simmons' equations with temperature factor in $\eta_{\mathrm{rect}} = [I_{\mathrm{dc}}(V_{\mathrm{b}} + \hbar\omega/e) + I_{\mathrm{dc}}(V_{\mathrm{b}} - \hbar\omega/e) - 2I_{\mathrm{dc}}(V_{\mathrm{b}})]/(\hbar\omega_{\mathrm{ex}}/e)^2$ to simulate the thermal effects on optical rectification. The barrier parameters used in the simulation are the same as those in Section S4. As the temperature increases from 300 K to 900 K, both the tunneling currents and the rectification currents increase, as shown in Figure S8. The trend of the tunneling currents and the rectification currents are the same as predicted above. For example, the trend of the rectification currents with V_{b} approaching 0 V is in an opposite way to the experimental results of the 3 nm Lu_2O_3 junction with 500 nm and 600 nm illuminations (Figure 5d, right two panels). This suggests that the thermal optical rectification and the normal thermal tunneling current do not dominate the thermal effects. Conversely, for the 3 nm Lu_2O_3 junction with 400 nm illumination, the thermal effects on optical rectification and the normal tunneling are dominant (Figure 5d, left panel).

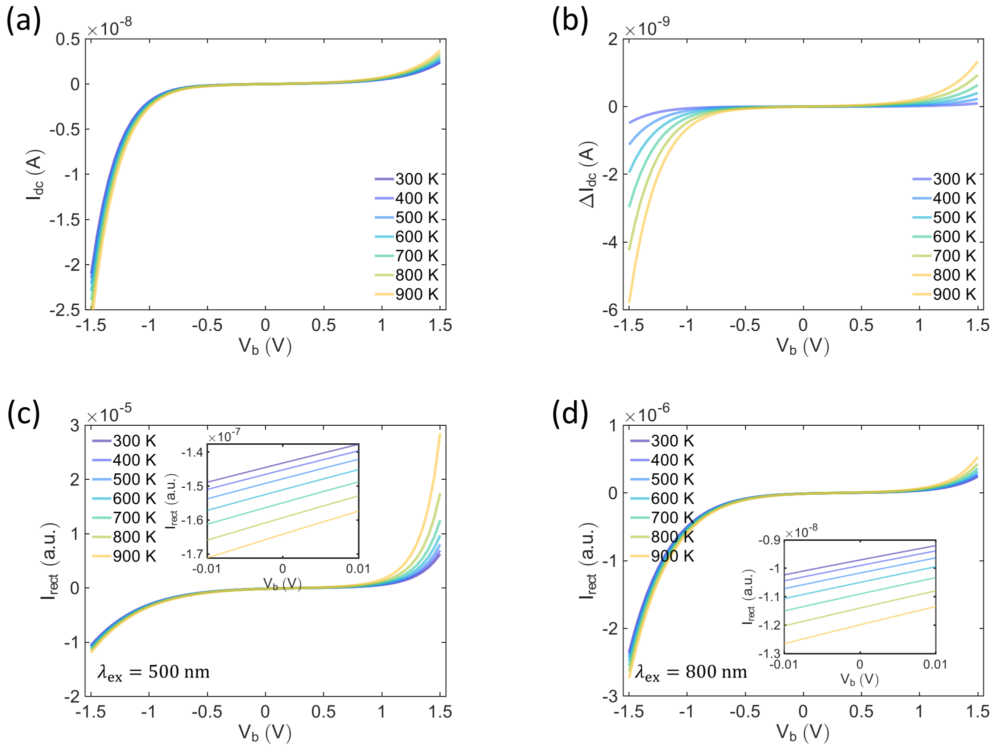

Figure S8. (a) I_{dc} as a function of V_{b} with the temperature varying from 300 K to 900 K. (b) $\Delta I_{\mathrm{dc}} = I_{\mathrm{dc}}(T) - I_{\mathrm{dc}}(T = 300\ \mathrm{K})$ as a function of V_{b} with the temperature varying from 300 K to 900 K.

I_{rect} as a function of V_{b} with the temperature varying from 300 K to 900 K under the 500 nm (c) and 800 nm (d) excitations. Insets: I_{rect} changes with temperature when V_{b} approaches to 0 V.

S8. Normalized Photon-to-current Responsivity

The normalized photon-to-current responsivity differences, $\overline{\Delta\eta_{\text{pc}}} = 2(-\eta_{\text{cp}}(-V_{\text{b}}) - \eta_{\text{pc}}(V_{\text{b}}))/2|-\eta_{\text{pc}}(-V_{\text{b}}) + \eta_{\text{pc}}(V_{\text{b}})|$, where $V_{\text{b}} > 0$, demonstrate the clear asymmetric properties of the junctions across the bias region and the wavelength range, (Figure S9a-c). As a result of varying η_{cp}, $\overline{\Delta\eta_{\text{pc}}}$ becomes larger with $|V_{\text{b}}|$ for $d \approx 2$ nm junction, which means higher negative V_{b} can provide higher efficiency (Figure S9a). $\overline{\Delta\eta_{\text{pc}}}$ is negative in the region $|V_{\text{b}}| < 0.2$ V due to the effects of hot electrons. For $d \approx 3$ nm junction, it is obvious that the turning point of η_{pc}, where $\eta_{\text{pc}}(V_{\text{b}}) > \eta_{\text{pc}}(-V_{\text{b}})$ depends on both V_{b} and λ_{ex}. Although $\overline{\Delta\eta_{\text{pc}}}$ has similar behavior for $d \approx 3$ nm junction when $\lambda_{\text{ex}} > 800$ nm (Region R1), $\overline{\Delta\eta_{\text{pc}}}$ becomes negative with $V_{\text{b}} < 1$ V when the illuminations are in the range of 500 nm $< \lambda_{\text{ex}} <$ 800 nm (Region R2), Figure S8b. Here, η_{rect} has not flipped with the increasing V_{b} yet because there is still $\overline{\Delta\eta_{\text{pc}}}(V_{\text{b}} > 1\text{ V}) > 0$. Obviously, the negative $\overline{\Delta\eta_{\text{pc}}}$ cannot be attributed to the change of η_{rect} but is the result of hot electron currents I_{hot} due to high enough photon energy. When $V_{\text{b}} > 1$ V, $\overline{\Delta\eta_{\text{pc}}}$ becomes positive and is dominated by η_{rect} again. However, η_{rect} is inverted with the $\lambda_{\text{ex}} = 400$ nm illumination, resulting in negative $\overline{\Delta\eta_{\text{pc}}}$ in the whole bias range (Region R3). For $d \approx 6$ nm junction, with the negligible I_{dc}, $\overline{\Delta\eta_{\text{pc}}}$ becomes negative in the whole region, where I_{hot} becomes comparable to I_{rect} or even dominates ΔI_{dc}.

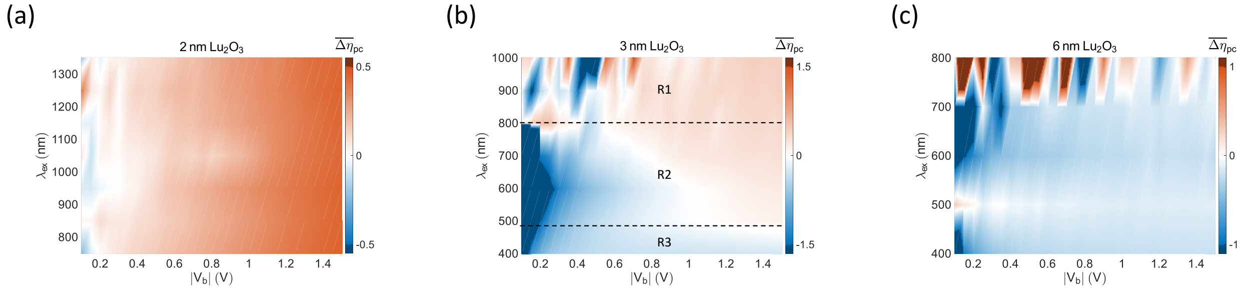

Figure S9. (a-c) The normalized photo-to-current efficiency differences, $\overline{\Delta\eta_{\mathrm{pc}}}$, as a function of $|V_{\mathrm{b}}|$ and λ_{ex} for the $d \approx 2$ nm, 3 nm, and 6 nm junctions, respectively.

S9. Power Dependence of the Laser-Induced Tunneling Current

For the $d \approx 2$ nm junction, we have shown the linear power dependence of ΔI_{dc} under the excitation wavelength varying from 750 nm to 1350 nm (Figure 3c). For the $d \approx 3$ nm junction, although there are some slightly nonlinear power-dependences of ΔI_{dc} when the excitation wavelengths approach the absorption peak (around 400 nm to 600 nm, Figure5c), the ΔI_{dc} is still dominated by linear process. To clarify the power-dependent properties of ΔI_{dc}, we use the equation $\Delta I_{\mathrm{dc}} = a \cdot P_{\mathrm{ex}}^{n}$ $(n \geq 1)$ to fit the data and analyze the power law.

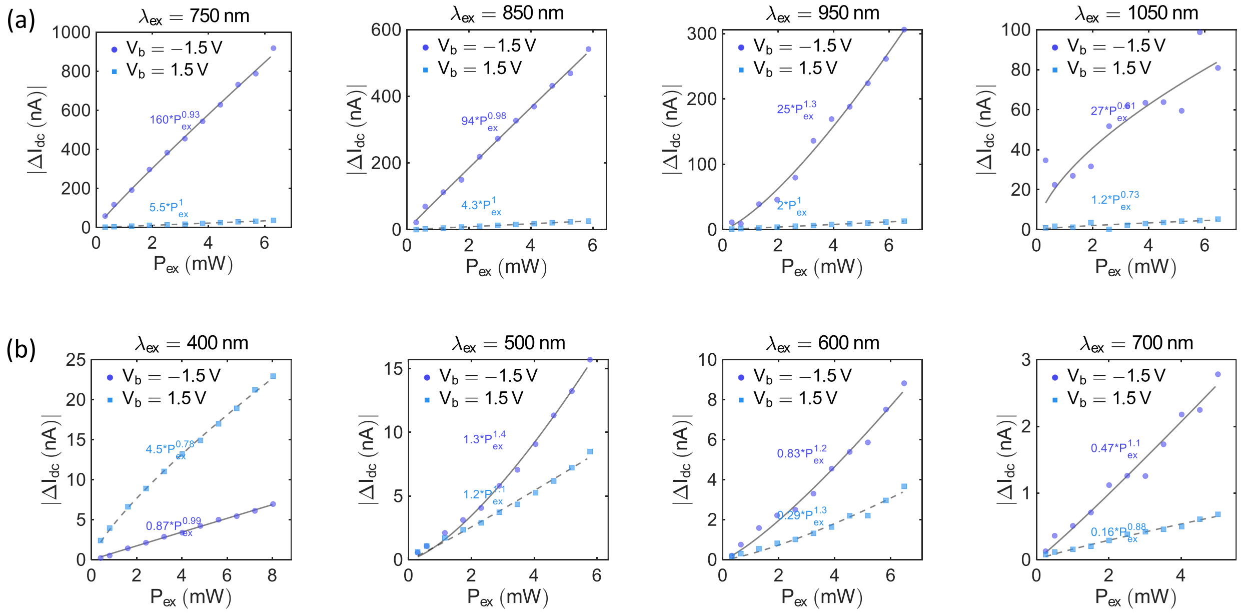

Figure S10. $|\Delta I_{dc}|$ as a function of P_{ex} for the $d \approx 2$ nm (a) and $d \approx 3$ nm (b) junctions. The purple circular dots represent the $|\Delta I_{dc}|$ with $V_b = -1.5$ V, and the solid lines are the fitting curves. The blue square dots represent the $|\Delta I_{dc}|$ with $V_b = 1.5$ V, and the dashed lines are the fitting curves.

It is obvious that most of ΔI_{dc} have a linear power dependence, Figure S10. Some ΔI_{dc} show slightly nonlinear behavior, with the extracted n slightly larger than 1. For instance, the $d \approx 2$ nm junction under 950 nm excitation and the $d \approx 3$ nm junction under 500 nm and 600 nm excitations. However, it is difficult to attribute this slight nonlinearity to the multi photon processes because their n remain well below 2. Some of the extracted n values are slightly smaller than 1. For instance, the $d \approx 2$ nm junction under 1050 nm excitation and the $d \approx 3$ nm junction under 400 nm excitation, which further suggests that the observed nonlinearities are unlikely caused by multi-photon effects. Taking into account the strong absorption observed around 400 nm to 600 nm (Figure 5b) and the corresponding abnormal changes in open-circuit voltage (Figure 5d), thermal effects are a more probable cause of the observed nonlinear power dependence.

S10. Laser-Induced Tunneling Current under CW-laser Excitation

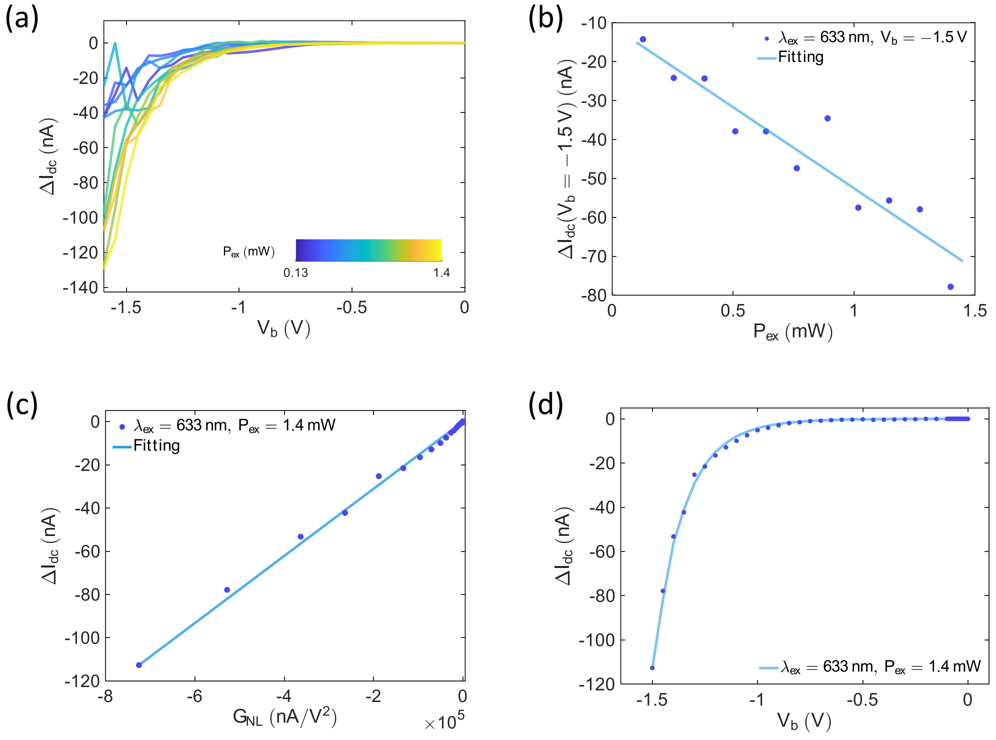

Figure S11. The $d \approx 2$ nm junction under 633 nm CW laser excitation. (a) ΔI_{dc} as a function of V_{b} with different excitation powers. (b) ΔI_{dc} as a function of P_{ex} with the bias $V_{\mathrm{b}} = -1.5$ V. (c) ΔI_{dc} as a function of G_{NL} extracted from the dark tunneling current with $P_{\mathrm{ex}} = 1.4$ mW. Dots: experimental results; line: fitting curve. (d) ΔI_{dc} as a function of V_{b} with $P_{\mathrm{ex}} = 1.4$ mW. Dots: experimental results; line: calculated results.

All experimental results for the optical rectification shown in the main text are measured under the excitation of femtosecond laser source. To examine the optical rectification using a CW laser illumination, we measure the $d \approx 2$ nm junction under 633 nm CW laser excitation and sweep the bias V_{b} from 0 to -1.6 V. It is obvious that there are no clear differences between the CW laser and the femtosecond laser excitations, as shown in Figure S11. As expected, ΔI_{dc} exponentially

increases with V_{b} and is proportional to P_{ex} under the CW laser illumination. Moreover, the ΔI_{dc} is linearly dependent on the nonlinearity of the conductance G_{NL} extracted via fitting the dark tunneling current, Figure S11c. As a result, the experimental data matches the calculated results (the product of the slop in Figure S10c and G_{NL}), Figure S11d.

S11. Effects of Asymmetric Barriers on Optical Rectification

As mentioned in the main text (Section 2.1), the optical rectification currents are proportional to optical rectification factor, $\eta_{\mathrm{rect}} = [I_{\mathrm{dc}}(V_{\mathrm{b}} + \hbar\omega/e) + I_{\mathrm{dc}}(V_{\mathrm{b}} - \hbar\omega/e) - 2I_{\mathrm{dc}}(V_{\mathrm{b}})]/(\hbar\omega_{\mathrm{ex}}/e)^2$. Thus, the asymmetric barriers have a significant effect on the rectification currents, I_{rect}. Here, we substitute I_{dc} with Eq. S1 in η_{rect} to explore the barrier asymmetry effects, Figure S12.

For the symmetric barrier, it is obvious that there is no I_{rect} at $V_{\mathrm{b}} = 0$ V because $I_{\mathrm{dc}}(V_{\mathrm{b}} + \hbar\omega/e)$ and $I_{\mathrm{dc}}(V_{\mathrm{b}} - \hbar\omega/e)$ compensate each other, shown in Figure S12c and f. When $\psi_1 \neq \psi_2$, the asymmetric barriers give raise to nonzero η_{rect} at $V_{\mathrm{b}} = 0$ V. The value of η_{rect} is mainly dependent on the average barrier height. Although the barrier of $\psi_1 = 1$ eV and $\psi_2 = 5$ eV (Figure S12a) is much more asymmetric than that of $\psi_1 = 1.5$ eV and $\psi_2 = 2.5$ eV (Figure S12e), I_{rect} of the former is much smaller than that of the latter due to the higher average barrier height of the former. Simultaneously, the photon energy also plays an important role in the optical rectification. Ideally, maximizing optical rectification factor, η_{rect}, can optimize the optical rectification current for the specific incident wavelength.

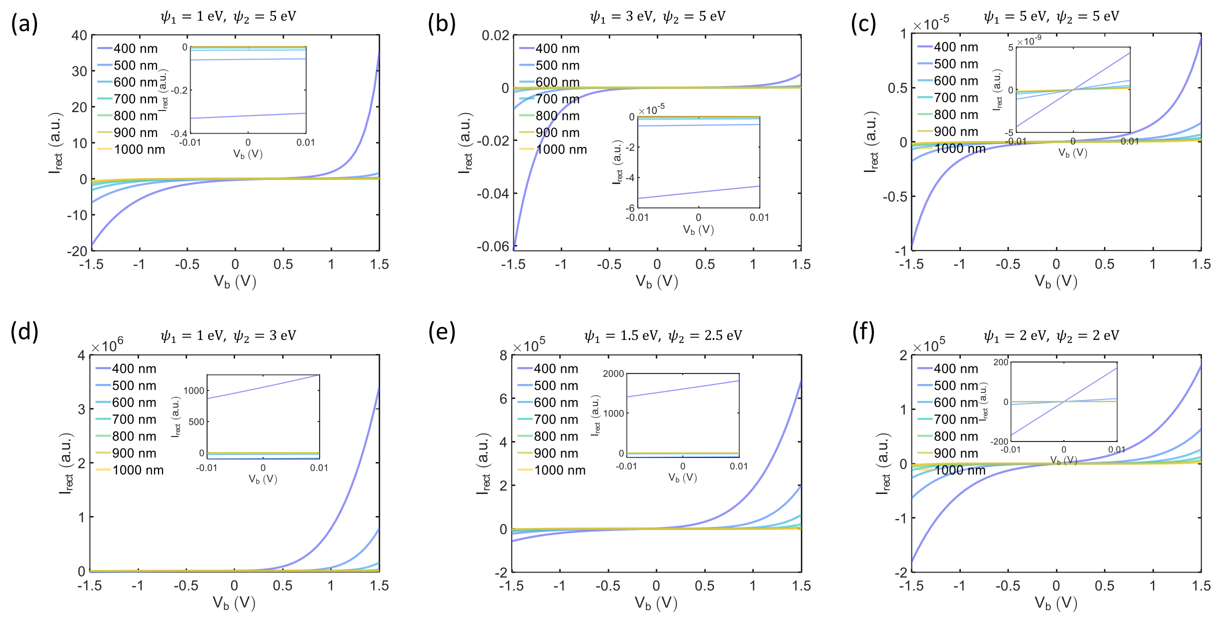

Figure S12. I_{rect} as a function of V_{b} under different incident wavelengths. (a-c) $\psi_2 = 5$ eV is fixed, ψ_1 changes from 1 eV to 5 eV, respectively. (d-f) $\psi_1 + \psi_2 = 4$ eV is fixed, ψ_1 changes from 1 eV to 2 eV, and ψ_2 changes from 3 eV to 2 eV, respectively. Insets: I_{rect} with V_{b} approaching 0 V.

S12. Electroluminescence (EL) in Tunnel Junctions

As discussed in Refs. [6, 7], the EL intensity is dependent on three main factors: inelastic electron tunneling (IET) rate, the local density of optical states (LDOS), and the outcoupling efficiency. Thus, for a specific junction with a fixed LDOS and outcoupling efficiency, the EL intensity should be proportional to the number of inelastic tunneling electrons. As expected, it should be proportional to the tunneling current as well.

For the $d \approx 2$ nm junction, the EL intensities shown in Figure 2d (main text) are quasi-linearly dependent on the tunneling currents under different V_{b}, as shown in Figure S13. Its electron-to-photon conversion efficiencies at different V_{b} are listed in Table S1. In this work, the junction

structure was not optimized and lower barrier material which would improve LDOS, outcoupling efficiency, and tunneling currents were not used. For instance, plasmonic structures can be integrated into the junction to enhance LDOS and amplify the outcoupling efficiency. Additionally, it should be noted that the collection efficiency is not taken into account due to the unknown EL emission pattern. Thus, the estimated conversion efficiencies should be lower than the actual values.

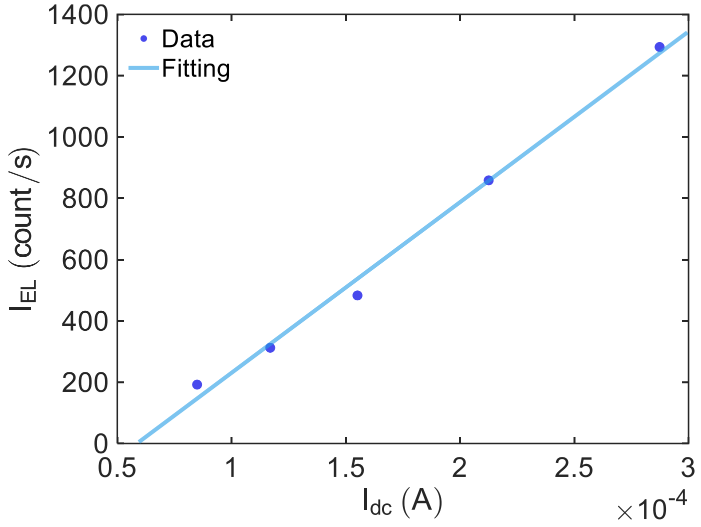

Figure S13. In the $d \approx 2$ nm junction, EL intensities, I_{EL}, as a function of tunneling currents at bias V_{b} =1.7 V, 1.75 V, 1.8 V, 1.85 V, and 1.9V. Dots: experimental data; line: fitting curve. Their EL spectra are shown in Figure 2d.

Table S1. Electron-to-Photon Conversion Efficiency

V_{b} (V)	Photon (count/s)	Electron (count/s)	η_{etp}
-1.90	1.29E+03	1.79E+15	7.21E-13
-1.85	8.58E+02	1.33E+15	6.47E-13
-1.80	4.83E+02	9.68E+14	4.99E-13
-1.75	3.12E+02	7.30E+14	4.28E-13
-1.70	1.92E+02	5.30E+14	3.62E-13

S13. Conversion Efficiency

The photon-to-electron conversion efficiency, η_{pte}, and the optical power-to-electrical power conversion efficiency, η_{power}, are shown in Figure S14. Their trend is similar to that of the photon-to-current responsivity, where a thinner Lu_2O_3 layer and a higher bias voltage have higher conversion efficiency. The highest photon-to-electron conversion efficiency around 4.78×10^{-4} and the highest optical power-to-electrical power conversion efficiency around 4.62×10^{-4} are both achieved in the 2 nm Lu_2O_3 junction with -1.6 V bias and 750 nm excitation.

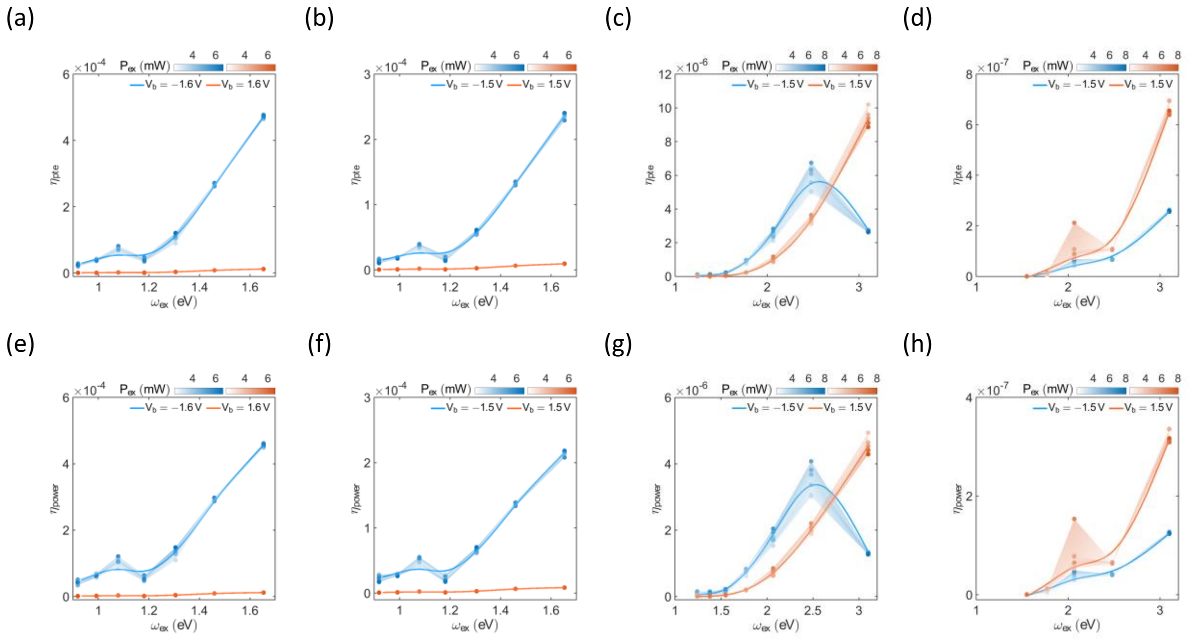

Figure S14. (a-d) Photon-to-electron conversion efficiency, η_{pte}, as a function of the incident photon energy, ω_{ex}, for the 2 nm (a-b), 3nm (c), and 6 nm (d) Lu_2O_3 junctions with different bias voltages and incident powers, respectively. (e-h) Optical power-to-electrical power conversion efficiency, η_{power}, as a function of the incident photon energy, ω_{ex}, for the 2 nm (e-f), 3nm (g), and 6 nm (h) Lu_2O_3 junctions with different bias voltages and incident powers, respectively.

S14. Stability of Tunnel Junctions

We evaluated the stability of a 3 nm Lu_2O_3 sample, as shown in Figure S15a. The sample was measured 154 times (77 measurements with laser illumination, 77 without) over 2 days with a total of 6 hours of bias voltage sweeps. The statistical analysis is based on the dark tunneling currents. Excluding low signal-to-noise regions, the RSD for 1 hour (11 measurements) is around 1.18%. With $V_\mathrm{b} < -0.5$ V, the RSD in 1 hour (11 measurements) is around 0.19% and 0.86% for the 2 nm and 3 nm Lu_2O_3 samples, respectively (Figure S15b and c). Additionally, we tested three additional Lu_2O_3 junctions over an extended period (more than one week, five measurements), as shown in Figure S15d-f. Their measured currents remain stable with RSD below 5%.

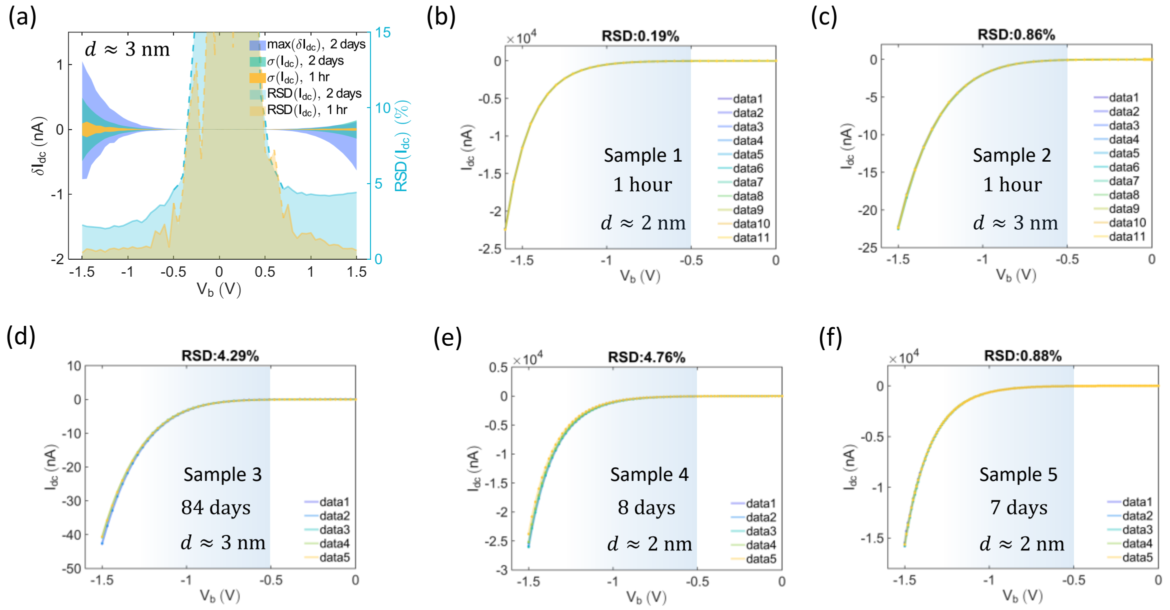

Figure S15. (a) Statistical data of the tunneling currents of the $d \approx 3$ nm junction. Left axis: The maximum current fluctuations $\max(\delta I_\mathrm{dc})$ as a function of V_b with regards to the average current in 2 days (purple shade). The current standard deviation $\sigma(I_\mathrm{dc})$ as a function of V_b (green shade: 2 days; yellow shade: 1 hour). Right axis: the relative standard deviation of I_dc (RSD), which is also called the coefficient of variation, of the currents as a function of V_b showing the variation of the

currents. The dash lines indicate the low signal-to-noise area, due to the limitation of the sourcemeter. (b-f) I_{dc} as a function of V_{b} for the junctions with different Lu_2O_3 thicknesses measured in different time periods. RSD are calculated for $V_{\mathrm{b}} < -0.5$ V.

S15. Characterization of the Epitaxially Grown Lu_2O_3 Junction

The cross-sectional images of the ITO/Lu_2O_3/Cr/Au are acquired via High-angle Annular Dark-field Scanning Transmission Electron Microscopy (HAADF-STEM), Figure S16. The individual layers are distinguishable, and the epitaxially grown ITO (bottom electrode) and Lu_2O_3 (insulation) layers show a clean coherent interface at higher magnification.

As shown in Figure S17, the XRD characterization of the epitaxially grown films on a YSZ substrate reveals that the (00ℓ) crystallographic planes of YSZ are parallel to the (00ℓ) planes of both ITO and Lu2O3 in the film, indicating good epitaxial alignment as expected. Furthermore, the rocking curves measured for the (004) Bragg peaks show a very low full width at half maximum (FWHM), providing additional evidence of the film's single-crystalline quality.

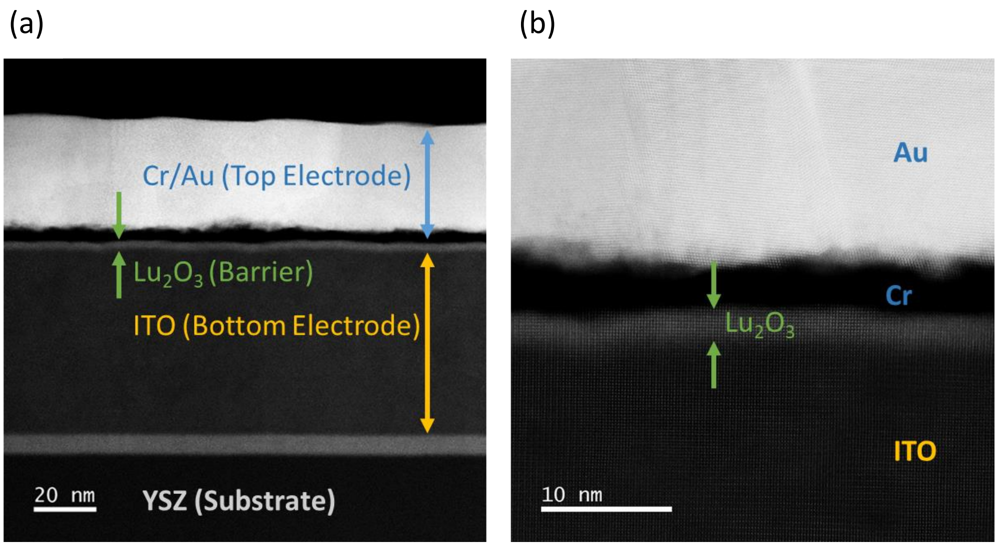

Figure S16. Cross-section HAADF-STEM images for the Lu_2O_3 junction (a) lower magnification and (b) higher magnification.

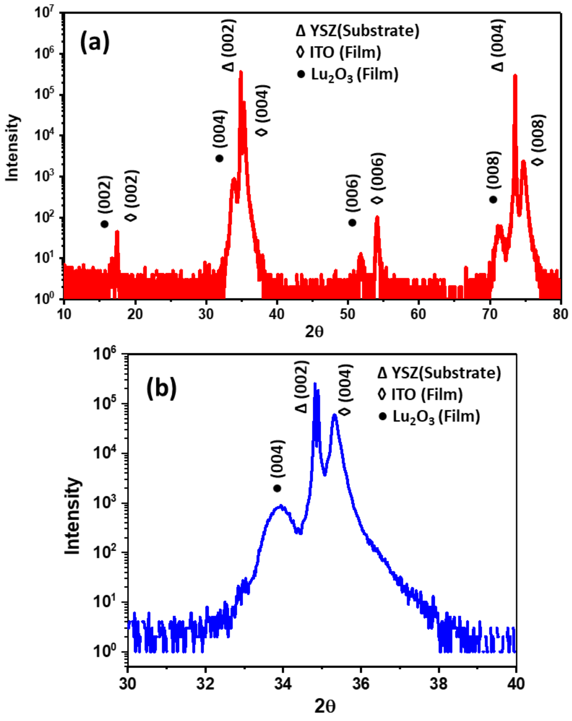

Figure S17. XRD characterization (a) θ-2θ scan and (b) high resolution θ-2θ scan for ~ 70 nm ITO and ~ 10 nm Lu_2O_3 film grown on YSZ (001) substrate.

REFERENCES

(1) Simmons, J. G. Generalized Formula for the Electric Tunnel Effect between Similar Electrodes Separated by a Thin Insulating Film. *J. Appl. Phys.* **1963**, *34* (6), 1793-1803. DOI: 10.1063/1.1702682.

(2) Simmons, J. G. Electric Tunnel Effect between Dissimilar Electrodes Separated by a Thin Insulating Film. *J. Appl. Phys.* **1963**, *34* (9), 2581-2590. DOI: 10.1063/1.1729774.

(3) Simmons, J. G. Generalized Thermal J-V Characteristic for the Electric Tunnel Effect. *J. Appl. Phys.* **1964**, *35* (9), 2655-2658. DOI: 10.1063/1.1713820.

(4) Hartman, T. E. Tunneling Through Asymmetric Barriers. *J. Appl. Phys.* **1964**, *35* (11), 3283-3294. DOI: 10.1063/1.1713211.

(5) Zolotavin, P.; Evans, C.; Natelson, D. Photothermoelectric Effects and Large Photovoltages in Plasmonic Au Nanowires with Nanogaps. *J. Phys. Chem. Lett.* **2017**, *8* (8), 1739-1744. DOI: 10.1021/acs.jpclett.7b00507.

(6) Parzefall, M.; Novotny, L. Light at the End of the Tunnel. *ACS Photonics* **2018**, *5* (11), 4195-4202. DOI: 10.1021/acsphotonics.8b00726.

(7) Parzefall, M.; Novotny, L. Optical Antennas Driven by Quantum Tunneling: a Key Issues Review. *Rep. Prog. Phys.* **2019**, *82* (11), 112401. DOI: 10.1088/1361-6633/ab4239.
